# An AR 3D Tracking and Registration Method That Integrates Optical Flow Tracking and Mean Shift

**DOI:** 10.3390/s26175509

**Published:** 2026-08-30

**Authors:** Jiu Yong, Xiaomei Lei, Jianwu Dang

**Affiliations:** 1 The School of Electronic and Information Engineering, Lanzhou Jiaotong Univeristy, Lanzhou 730070, China; 2 School of Computer Science, Peking University, Beijing 100871, China; 3 College of Intelligence and Computing, Tianjin University, Tianjin 300072, China

**Keywords:** augmented reality, 3D registration, feature matching, target tracking, optical flow tracking, mean shift

## Abstract

**Highlights:**

- A confidence weighted feature matching and homography estimation strategy is proposed to improving inlier selection and pose estimation accuracy.
- A robust Augmented reality (AR) 3D tracking registration is achieved based on optical flow tracking and mean shift.

**Abstract:**

Augmented reality (AR) enhances the real world scene by overlaying virtual information onto it. Vision-based 3D tracking and registration is the key technology for ensuring the fusion of virtual and real content in monocular AR systems. Existing mainstream visual tracking and registration methods are susceptible to illumination variations, motion blur, target occlusion, and dynamic background interference in complex scenarios. They also suffer from low computational efficiency, cumulative pose errors, and insufficient stability, making them difficult to deploy on low power edge devices such as embedded systems and mobile terminals. To address these issues, this paper proposes a lightweight monocular AR 3D tracking and registration method that integrates ORB-FREAK features, mismatching outlier filtering, background weighted mean shift, and template-based relocalization. The method does not rely on depth sensors or neural network inference, enabling efficient and accurate lightweight pose estimation. Specifically, we first combine the ORB (Oriented FAST and Rotated BRIEF) descriptor with the FREAK (Fast Retina Keypoint) algorithm for feature detection and initial matching. Hamming distance is used for coarse filtering of mismatched point pairs, and an ascending sort combined with an iterative sequential sampling strategy is applied to solve the optimal homography matrix, significantly improving the accuracy and efficiency of matrix estimation. Then, distance constraints among feature points are imposed on the target registration region to optimize the selection, and camera pose is computed based on the matching between 2D feature points and their corresponding 3D spatial coordinates, eliminating the error accumulation problem of conventional algorithms. Real-time feature matching is further used to correct the optical flow tracking sequence and camera pose, ensuring the continuity of the AR tracking process. Finally, a background weighted mean shift algorithm is introduced to narrow the feature detection range and suppress background interference, complemented by a template-matching relocalization module and a dynamic model update strategy, which effectively enhance the robustness of continuous tracking and registration under complex conditions. Experimental results demonstrate that, in extreme scenarios such as low light conditions, high speed motion, and occlusion, the proposed method achieves AR 3D tracking and registration success rates of 86.7%, 82.3%, and 78.5%, respectively. It exhibits superior performance in pose estimation accuracy and anti-interference capability in complex environments, with significantly reduced computational overhead. Moreover, it can achieve robust and continuous AR 3D tracking and registration on low power edge devices, effectively adapting to demanding AR application scenarios and providing reliable technical support for lightweight AR applications.

## 1. Introduction

Augmented reality (AR) can superimpose virtual information such as 3D models, images, and text onto the real environment in real time, effectively compensating for human perception of real scenes and enhancing human–computer interaction [1]. Owing to its immersive, visual, and virtual–real fusion characteristics, AR technology has been deeply applied in various fields including industrial maintenance, clinical medicine, education and teaching, and cultural tourism [2]. 3D tracking and registration, virtual–real fusion display, and real-time virtual–real interaction are the three key technologies of AR systems, among which 3D tracking and registration is the fundamental prerequisite for achieving virtual–real fusion and dynamic interaction [3]. This technology performs real-time estimation of the camera’s position and orientation in the real scene to achieve precise alignment and overlay of virtual models with the real environment. Its registration accuracy, real-time performance, and stability directly determine the overall operational performance of AR systems and are the key to the innovative deployment of AR technology [4]. Currently, computer vision-based AR 3D tracking and registration technology has become the mainstream research direction in the industry, owing to its low hardware cost, easy deployment, and strong adaptability. This technology relies on inherent feature information of the real scene for camera pose estimation, without the need for additional sensing devices, and possesses a certain degree of anti-interference capability against common complex scenarios such as partial target occlusion and boundary out-of-view [2]. However, existing mainstream visual feature-based tracking and registration methods still have significant technical shortcomings, such as insufficient feature matching accuracy, poor long-term tracking stability, and weak adaptability to complex scenes. They struggle to meet the high-precision real-time registration requirements in dynamic and complex environments, and thus urgent improvement is needed.

The current mainstream workflow for 2D planar target visual-feature-based AR 3D tracking and registration mainly includes scene information detection and recognition, camera pose estimation, virtual information rendering and fusion, and scene output display [5,6]. According to the different ways of feature acquisition, this technology can be divided into two categories: artificial marker-based and natural marker-less [7]. Among them, artificial marker tracking methods are highly susceptible to environmental disturbances such as illumination, occlusion, and cluttered backgrounds, resulting in poor registration stability and limited application scenarios [8]. In contrast, marker-less tracking methods rely on natural features such as corner points, edges, and contours inherent in the scene for pose estimation, without the need for preset markers, offering wider scene adaptability and currently being a research hotspot in AR tracking and registration. However, marker-less algorithms are highly sensitive to environmental changes, including scale variation, viewpoint shifts, and brightness fluctuations, which increase the difficulty of feature extraction and matching and impose high demands on real-time performance and robustness [9]. Moreover, although deep learning-based AR tracking and registration methods have achieved some success in recent years, their large model parameters and high computational complexity make them difficult to adapt to lightweight, low-computing-power application scenarios on mobile devices [10]. Therefore, existing visual-feature-based AR 3D tracking and registration methods generally suffer from low computational efficiency, weak robustness in complex environments, and insufficient tracking stability, which severely restrict the large-scale application and promotion of AR technology.

To address the above problems, this paper proposes an AR 3D tracking and registration method that integrates optical flow tracking and mean shift algorithms. By optimizing the feature matching mechanism, pose estimation strategy, and target tracking approach, the method improves the real-time performance, stability, and robustness of camera pose estimation, achieves high-precision and sustained virtual–real fusion between virtual information and the real scene, effectively adapts to complex AR application scenarios, and provides reliable technical support for lightweight AR applications. The main contributions of this paper are as follows:

1. A confidence weighted feature matching and homography estimation strategy is proposed. By adopting a Hamming distance based ascending ordered sampling iterative mechanism, the efficiency of homography matrix solution is improved. A multi-path confidence model for matching is constructed by integrating matching similarity, motion consistency, and geometric constraints, combined with clustering-based pre-filtering to eliminate outliers, effectively resisting dynamic foreground interference, improving inlier selection and pose estimation accuracy, and enhancing the generalization performance of the algorithm in scenarios with drastic illumination and scale changes.
2. A threshold-adaptive tracking perception and pose recovery module is designed to address the problems of long-term drift, easy interruption, and poor adaptability of fixed parameters in traditional optical flow tracking. A 2D–3D region constrained pose estimation model is built to correct optical flow tracking deviations in real time. Combined with image signal to noise ratio and feature density, a quantifiable adaptive tracking discrimination model is constructed, which can perceive feature degradation and trigger relocalization in complex scenarios such as low light and motion blur, significantly improving tracking robustness in complex scenes.
3. An improved CBWH (Centroid-based background weighted Histogram) mean shift and layered foreground constraint strategy is integrated. By dynamically shrinking the feature search range to suppress dynamic background interference, combined with dynamic updating and intelligent relocalization modules, a closed-loop tracking architecture is formed to solve the problems of long-term tracking drift and loss, achieving lightweight anti-interference AR 3D tracking and registration. This breaks through the device deployment bottleneck of traditional AR algorithms and effectively adapts to mobile and embedded low-computing-power scenarios.

The organization of this paper is as follows: Section 2 reviews the current research status of AR 3D tracking and registration technology at home and abroad, summarizing the advantages and disadvantages of existing techniques; Section 3 elaborates on the overall framework and main module principles of the proposed method; Section 4 verifies the algorithm performance from multiple dimensions such as real-time performance, robustness, and accuracy through several comparative experiments; Section 5 analyses the innovations and existing limitations of this method; Section 6 summarizes the research findings and discusses future optimization and research directions.

## 2. Related Work

AR 3D tracking and registration is the key to achieving precise virtual–real fusion. By estimating the camera’s pose parameters relative to the real world target in real time, it accomplishes accurate overlay and rendering of virtual models, which is essential for high-precision virtual–real integration [11]. Currently, mainstream vision-based AR tracking and registration methods primarily rely on natural feature matching. They extract image keypoints and feature descriptors, match them against offline-built natural feature templates, and then solve for the relative pose between the camera and the registered target using geometric transformation relationships, ultimately achieving stable 3D tracking and registration [12]. Such methods can effectively exploit the inherent low-level features of images and exhibit good adaptability to transformations such as rotation, scaling, viewpoint shifts, and illumination variations, and have therefore been widely applied in various AR scenarios. Numerous studies have focused on optimizing the inherent drawbacks of traditional handcrafted features. For example, Hong et al. [13] proposed an ICP registration algorithm based on distance-constrained angular matching, achieving accurate point-cloud registration between real-scene objects and digital models, effectively enhancing the ability to represent scene information and resist environmental interference. However, these methods still suffer from feature matching failure, pose drift, and tracking interruption under conditions such as dynamic occlusion, severe illumination changes, and complex dynamic backgrounds.

Traditional frame-based camera 3D tracking methods are susceptible to extreme illumination conditions such as strong light exposure and drastic brightness changes. The limited dynamic range of sensors often leads to image saturation or underexposure, resulting in feature loss and tracking drift, making them poorly suited for extremely complex scenarios. To address this, two representative hardware-based optimization approaches have emerged in the field of complex-scene perception in recent years. The first is optical adaptive modulation technology based on Digital Micromirror Devices (DMD), which achieves high-dynamic-range imaging under strong glare through pixel-level light intensity control. This suppresses highlight saturation at the source, providing high-quality raw image input for 3D tracking and significantly reducing the probability of feature extraction failure [14]. The second is asynchronous perception technology, primarily represented by event cameras, which rely on an asynchronous brightness-change triggering mechanism. These cameras offer an ultra-high dynamic range and microsecond-level temporal resolution, making them suitable for tracking 3D deformations of high speed targets under extreme lighting. Through event-frame feature fusion and long-term state modelling, they can stably output 3D feature trajectories under severe illumination fluctuations, effectively compensating for the limitations of traditional frame-based cameras in high speed and strong-light scenarios [15]. Although these specialized sensing hardware can effectively improve the quality of image input in extreme scenes, existing methods generally suffer from high hardware costs, complex system integration, and difficult deployment, making them unsuitable for the widespread use of lightweight monocular AR devices [16,17]. This paper conducts research based on ordinary visible-light imaging equipment, aiming to improve tracking stability under complex illumination by optimizing robust feature matching and geometric constraint tracking strategies, without adding complex optical hardware, thereby complementing existing hardware-enhanced approaches. In addition, some studies leverage Convolutional Neural Networks (CNNs) to extract heterogeneous deep features from images, overcoming the limited representational capacity of traditional handcrafted features [18]. Park et al. [19] applied Mask R-CNN to RGB-D images, achieving precise AR registration on mobile devices through contour instance segmentation and point-cloud extraction. Li et al. [20] used a heatmap FCN model for registration in AR assembly scenarios and reduced dataset construction costs via virtual–real image fusion. Zhang et al. [21] built a hybrid point-set AR registration model, demonstrating outstanding performance in registration accuracy, convergence speed, and noise resistance. Li et al. [22] designed AR-Centernet, a network that integrates AR registration and target occlusion handling. Zaman et al. [23] employed a continuous graph attention network with knowledge distillation and retention mechanisms to achieve high-precision AR point-cloud registration. Although deep learning-based methods exhibit significant advantages in registration accuracy, they generally suffer from large model sizes and high computational complexity, relying heavily on high-performance hardware and making them difficult to adapt to lightweight, real-time AR applications on mobile devices.

Currently, AR 3D tracking and registration technologies for complex dynamic scenes mainly follow three approaches: RGB-D SLAM multi-sensor fusion, deep learning feature enhancement, and high-precision calibrated geometric modelling localization. For instance, Wu et al. [24] proposed a SLAM tracking framework integrating an RGB-D depth camera, which uses depth information to distinguish dynamic foreground objects from static backgrounds and combines dense-map constraints to improve tracking robustness in complex indoor and low-texture environments. However, this method heavily relies on dedicated depth sensors, incurring high hardware costs and large device sizes, making it unsuitable for lightweight mobile and monocular AR deployment, and is only applicable to fixed indoor scenes. Zhang et al. [25] enhanced feature detection and matching via neural networks, leveraging convolutional networks to mine deep semantic features, effectively alleviating matching failures caused by sudden illumination changes and slight occlusions. Nevertheless, the real-time performance of deep learning models degrades on embedded low power devices, failing to meet the demands of long-term continuous outdoor tracking. Will et al. [26] focused on high-precision 3D localization, designing a multi-stage camera calibration and detailed geometric modelling process to reduce pose estimation errors through precise intrinsic/extrinsic parameter correction and point-cloud registration modelling. This approach achieves excellent localization accuracy, but the calibration procedure is cumbersome, requiring recalibration when scene scale or viewpoint undergoes large changes, resulting in weak generalization and dynamic adaptability. It can be seen that all three approaches have inherent technical bottlenecks and cannot simultaneously satisfy the AR application requirements of low cost, low computational power, high robustness, and strong generalization. Additionally, visual SLAM technology, with its unique advantages in simultaneous localization and mapping, can build 3D maps of the scene in real time, freeing AR registration from reliance on offline prior feature databases [27,28] and effectively expanding the applicable scenarios of AR registration. However, in highly dynamic and heavily interfered environments, this technology is prone to feature loss, localization drift, and mapping failures, resulting in poor continuity and stability of tracking and registration, which cannot meet the demands of high-precision, sustained AR virtual–real interaction. It is evident that current mainstream natural-feature AR registration methods often combine visual features from RGB-D cameras with depth features to establish spatial pose constraints for improved registration robustness [29]. However, depth cameras have hardware shortcomings such as low acquisition frame rates and limited viewing angles, making it difficult to effectively recognize weakly visible or dynamically moving registration targets. Deep learning registration methods, on the other hand, overly rely on large-scale labelled dataset training and offline prior models, lacking scene adaptability and real-time capability on devices, thus failing to cope with complex dynamic scenes.

In summary, existing AR 3D tracking and registration technologies generally suffer from strong hardware dependence, high computational overhead, weak scene generalization, and insufficient robustness in extreme environments. There is a lack of lightweight and stable registration methods that can adapt to highly complex scenes and be deployed on low power edge devices. To this end, this paper focuses on AR 3D tracking and registration for 2D planar targets using an ordinary monocular visible-light camera, without relying on depth sensors or deep learning inference models. By optimizing the ORB-FREAK joint feature extraction, Hamming distance based coarse mismatching filtering, improved mean shift target region constraint, and a multi-module collaborative strategy including optical flow and template-based relocalization, we construct a lightweight, high-precision geometric-constraint-based pose estimation algorithm. This effectively circumvents the hardware dependency of multi-sensor methods, the high computational cost of deep learning approaches, and the generalization issues of traditional calibration methods. While ensuring pose estimation accuracy, it also accommodates the real-time deployment requirements of embedded and mobile low power devices, providing reliable technical support for the deployment of lightweight AR applications in complex scenarios.

## 3. Methods

**Figure 1:**
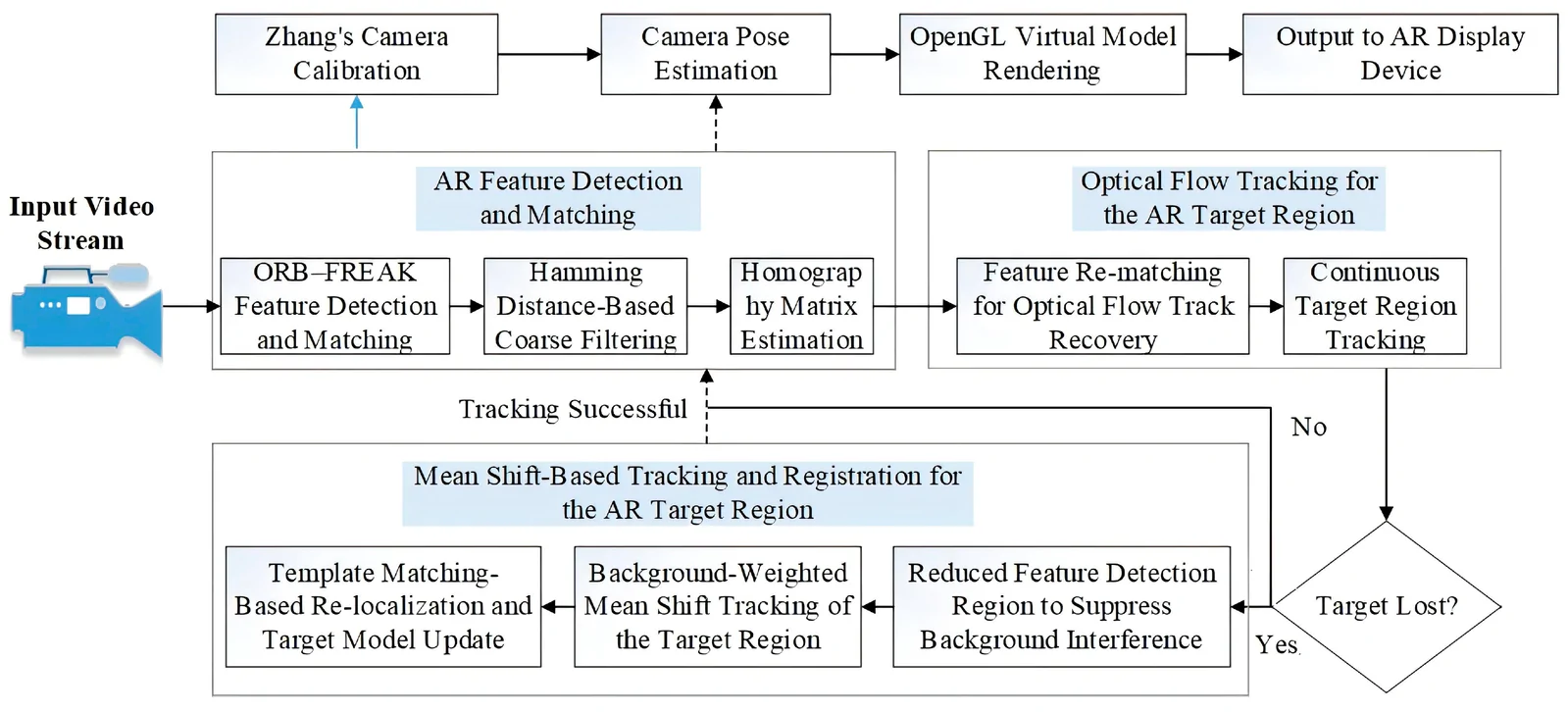
Method.

This paper proposes an AR 3D tracking and registration method that integrates optical flow tracking and mean shift. Through feature detection, matching, and tracking algorithms, the method processes the real-scene video stream captured by the camera, estimates the relative pose of the AR camera in the real environment, determines the position and orientation of the virtual model based on camera imaging principles and calibration techniques, and accomplishes precise fusion of the virtual model with the real scene via graphics rendering, with the final scene output presented through AR display devices. As shown in Figure 1, we combine the ORB and FREAK algorithms for feature detection and matching, balancing accuracy and real-time performance. An improved RANSAC algorithm is adopted to optimize the homography matrix solution. Relying on the proposed feature point selection, 2D–3D point matching, and pose recovery modules, we use real-time feature matching to correct the optical flow tracking sequence and camera pose deviations. The background weighted mean shift algorithm is introduced for target region tracking, and template-based relocalization and model update strategies are incorporated to enhance the stability of sustained registration. Ultimately, the proposed method accomplishes AR 3D virtual–real tracking and registration, achieving precise and real-time fusion of virtual models with the real scene.

### 3.1. AR Feature Detection and Matching

Robust AR feature point detection and matching are critical to the accuracy, real-time performance, and stability of 3D tracking registration in augmented reality. The more distinctive the intrinsic characteristics of the extracted feature points, the more beneficial they are to subsequent feature matching. Through feature point matching, the position and orientation of the camera relative to the real scene can be estimated, enabling accurate rendering of virtual models; thus, the matching accuracy directly affects the registration precision. Currently, mainstream feature point detection algorithms include SURF, AKAZE, ORB, and FREAK [30]. In this paper, the ORB algorithm is primarily adopted for feature point extraction, combined with the FREAK binary descriptor for feature matching. An improved RANSAC algorithm is then employed for match purification, and the optimal homography matrix is solved.

#### 3.1.1. ORB Combined with FREAK for Feature Point Extraction and Description

**Figure 2:**
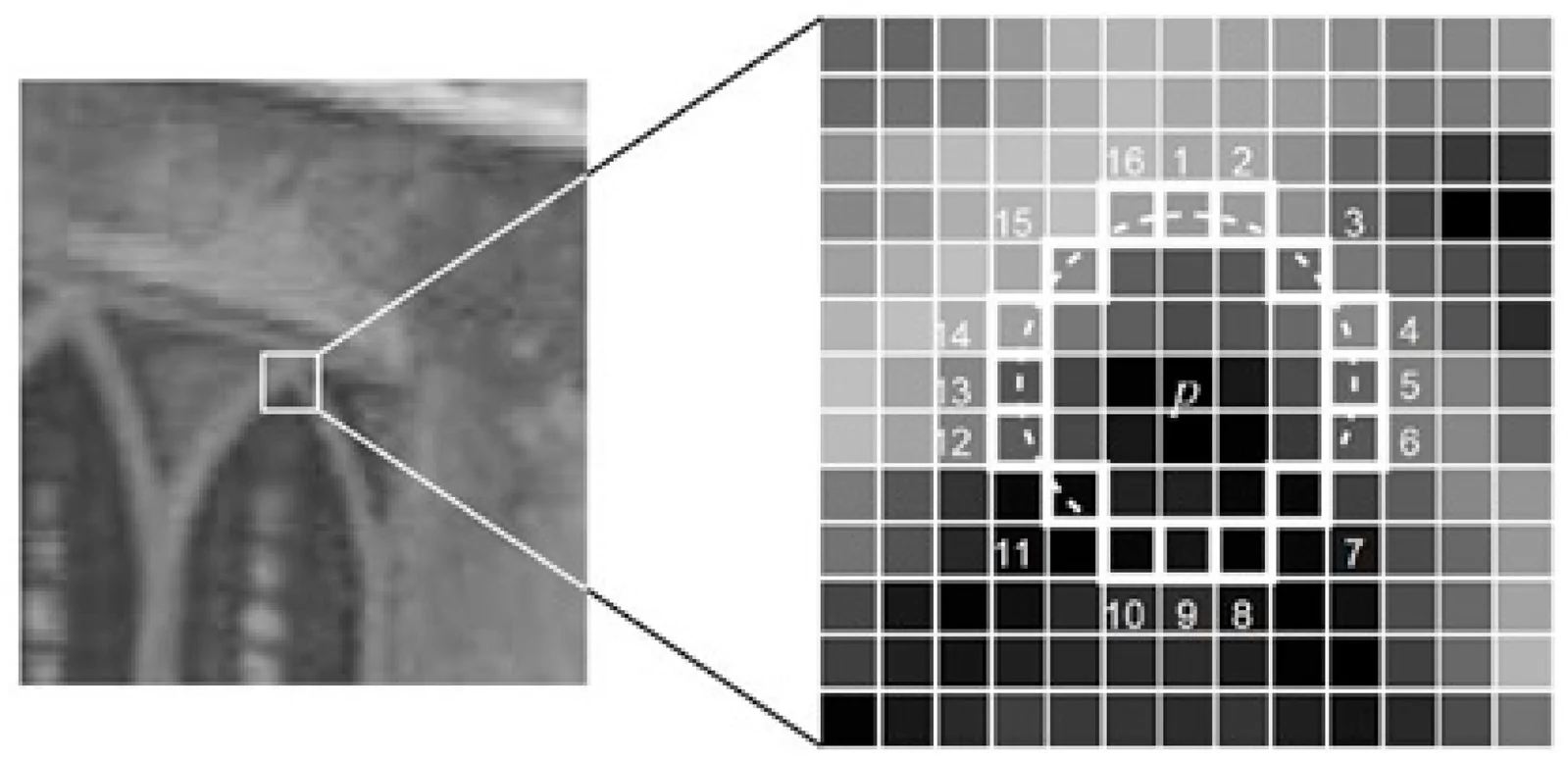
FAST feature point detection.

The ORB algorithm employs an improved FAST algorithm for feature point extraction. First, FAST feature detection is performed on the image. As shown in Figure 2, a circle of radius *r* is constructed around a pixel *p* in the target image. If there exist *t* consecutive pixels $q_{i}$ on the circumference that satisfy Equation (1), then pixel *p* is determined to be a feature point:

(1) $$\left|I\left(p\right)-I\left(q_{i}\right)\right|>\gamma$$

Among them, $I\left(p\right)$ denotes the grayscale value of the pixel at the center of the circle, and $I\left(q_{i}\right)$ represents the grayscale value of any pixel on the circumference.

An image pyramid is constructed by continuously down-sampling with a scale factor of 1.2, and the number of pyramid levels is set to 8. FAST feature detection is performed on the images at different pyramid levels, and all detected feature points are sorted using the Harris response function, retaining the top *N* points with the largest response values. The positions of feature points with scale invariance are then obtained by fitting via two-dimensional quadratic interpolation in the image space and one-dimensional interpolation in the adjacent scale space.

**Figure 3:**
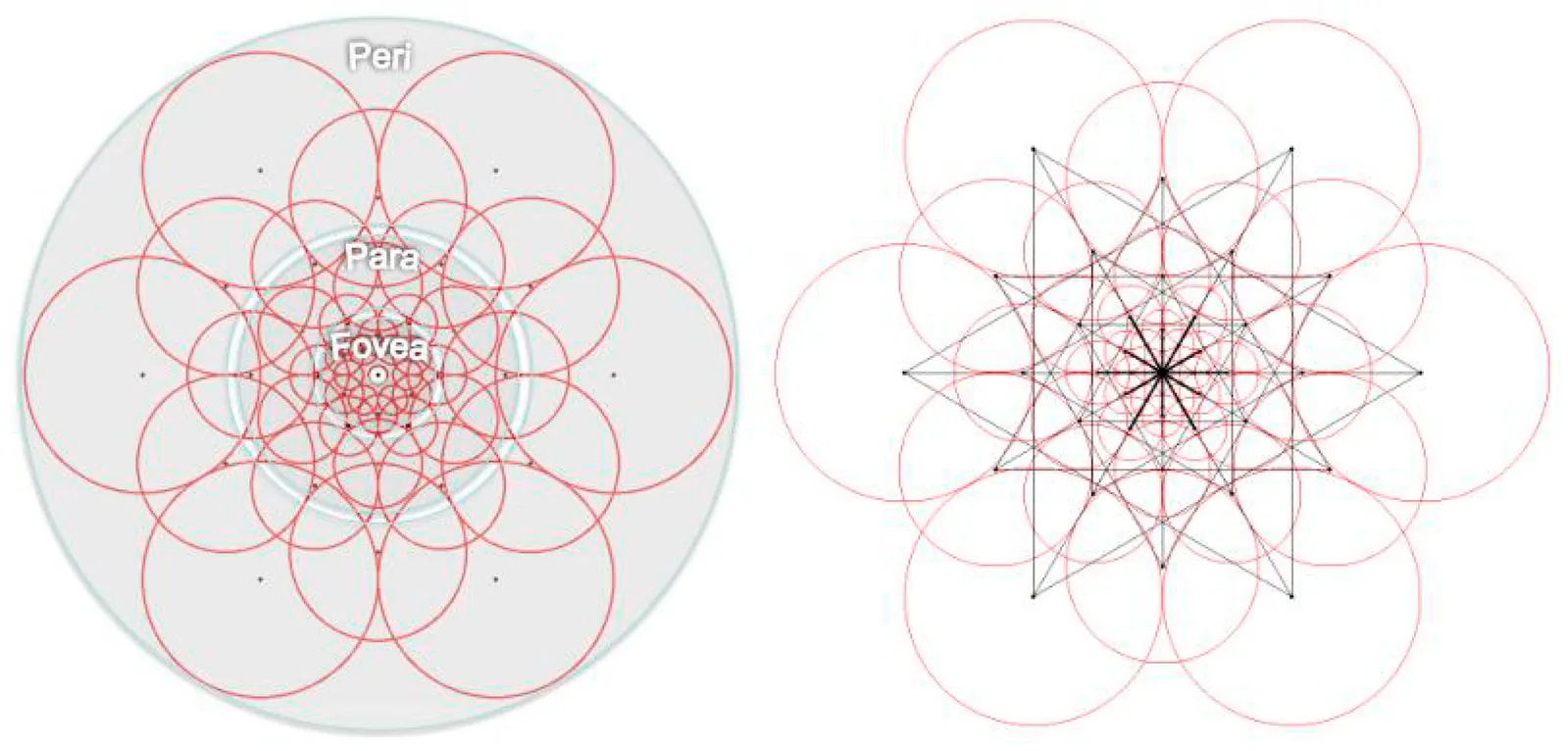
FREAK sampling pattern.

FREAK is a binary feature descriptor that adopts a sampling model closer to the way the human retina receives image information. The sampling-point structure is shown in Figure 3. A circular structure with different Gaussian kernel standard deviations is established around each sampling point, and Gaussian blurring is applied to each sampling point to provide a certain degree of noise robustness. The centermost point is the feature point; the farther a sampling point is from the feature point, the larger the Gaussian blur radius. Because this sampling pattern causes the receptive fields of adjacent sampling points to overlap, more information is captured under this structure, which improves the distinctiveness of the descriptor.

Generate a series of binary codes by comparing the grayscale values of sampled point pairs, denoted as *F*:

(2) $$F=\sum_{0\leq a\leq N}2^{a}T\left(P_{a}\right)$$

(3) $$Q\left(P_{a}\right)=\left\{\begin{array}{cc} 1 & if\left(I\left(P_{a}^{r^{1}}\right)-I\left(P_{a}^{r^{2}}\right)\right)>0,\\ & 0\;otherwise, \end{array}\right.$$

Among them, $P_{a}$ represents any pair of sampling points, and *N* denotes the number of sampling point pairs, which is also the length of the binary descriptor, which is the length of the binary descriptor. $I\left(P_{a}^{r^{1}}\right)$ and $I\left(P_{a}^{r^{2}}\right)$ represents the Gaussian smoothed grayscale values of the sampling points. If the difference in grayscale values is greater than 0, the corresponding position of the descriptor will be set to 1, otherwise it will be set to 0.

A coarse-to-fine retinal saccadic matching search is adopted, in which sampling pairs in the outer ring are selected first, followed by those in the inner ring. The first 128 bits of the two descriptors to be matched are compared; if the result is below a preset threshold T, the subsequent 128 bits of the two descriptors are then matched. A set of 45 symmetric sampling point pairs is selected, and the average of their local gradients is computed to assign orientation information to the descriptor. The gradient is:

(4) $$O=\frac{1}{M}\sum_{P_{o}\in G}\left(I\left(P_{o}^{r_{1}}\right)-I\left(P_{o}^{r_{2}}\right)\right)\frac{P_{o}^{r_{1}}-P_{o}^{r_{2}}}{\left\|P_{o}^{r_{1}}-P_{o}^{r_{2}}\right\|}$$

Among them, *M* represents the total number of sampling point pairs, *G* represents the selected set of sampling point pairs, $P_{o}^{r_{1}}$ and $P_{o}^{r_{2}}$ are the coordinates of the sampling points.

#### 3.1.2. RANSAC Algorithm for Match Purification

To further optimize the quality of feature matching, this paper employs the RANSAC algorithm for match purification. The RANSAC algorithm estimates an initial homography matrix by random sampling, then iteratively refines the estimation to obtain the optimal result and the corresponding homography matrix. The more false matches in the initial matching set, i.e., the lower the inlier ratio, the more unfavorable it is for the iterative computation of the optimal homography matrix, and the higher the required number of iterations tends to be. The similarity of feature point pairs can be applied to the sample selection of LMedS, and combined with the inherent property of RANSAC that adaptively adjusts the iteration count based on the number of inliers, this prevents premature termination of iterations and ensures that the optimal homography matrix is not missed.

(1) **Coarse filtering of some false matches using known Hamming distance**

The Hamming distance is used to measure the similarity between binary descriptor vectors. It is defined as the total number of bit positions at which the corresponding bits differ between two binary vectors of equal length, i.e., the number of bit substitutions required to transform one vector into the other. The higher the vector similarity, the smaller the Hamming distance. Thus, the Hamming distance can be used to evaluate the similarity of feature point matches. The classical screening strategy typically adopts the constraint that the Hamming distance is less than twice the minimum Hamming distance to filter matches. In this paper, after multiple sets of comparative tests, a Hamming distance threshold of 100 is selected as the coarse-filtering criterion. When the Hamming distance of a match pair exceeds 100, the match pair contains a large number of false matches; this threshold achieves the best matching-filtering performance on the current dataset. Note that this threshold is a dataset-dependent empirical parameter and is not universally applicable. When transferring to other datasets, parameter tuning should be performed according to the descriptor bit length, image noise level, and feature texture richness. A grid-search approach can be used to traverse candidate thresholds, with matching accuracy and inlier ratio as evaluation metrics to determine the optimal value. The matches filtered by the Hamming distance are then fed into the subsequent RANSAC algorithm for geometric constraint verification. In addition, to avoid solving errors in 2D–3D pose estimation caused by excessive clustering of feature points, this paper introduces a spatial distance constraint to filter candidate feature points. This constraint is applied simultaneously to both the point set output by optical flow tracking and the feature point set obtained by matching. The distance constraint controls the minimum pixel interval between feature points, filters out spatially over-close features, ensures a uniform distribution of feature points within the target region, and improves the numerical stability of pose estimation. Let the pixel coordinates of two feature points be $p_{i}(u_{i},v_{i}),p_{j}(u_{j},v_{j})$. The Euclidean pixel distance is computed as:

(5) $$d_{ij}=\sqrt{{\left(u_{i}-u_{j}\right)}^{2}+{\left(v_{i}-v_{j}\right)}^{2}}$$

Distance constraint criterion: if $d_{ij}<d_{th}$, then one of the neighboring feature points is removed. $d_{th}$ is the minimum spatial distance threshold. The algorithm adopts a non-maximum suppression greedy selection strategy: all detected features are sorted in descending order of their Harris corner response values, with higher-response corners retained preferentially. The remaining candidate points are then traversed; if the distance between a candidate and any point already in the retained set is less than $d_{th}$, the candidate is discarded directly. The final output is a feature set that is relatively uniformly distributed in space.

(2) **Sorting by similarity and selecting samples sequentially**

Data points with higher similarity are more likely to be inliers. The smaller the Hamming distance, the higher the similarity of any matching pair. Therefore, based on the Hamming distances of the remaining matching pairs, the distances can be sorted in ascending order. Samples are selected sequentially as $\left\{1,2,3,4\right\},\left\{5,6,7,8\right\},\cdots ,$$\left\{4m+1,4m+2,4m+3,4m+4\right\}$, where *m* is the total number of iterations of the algorithm. An initial homography matrix is estimated from the sequence of points selected in each order, and the remaining matching points are tested. Finally, the optimal homography matrix and the inlier set are computed, completing the feature point matching.

Therefore, by adopting the ORB algorithm for feature point extraction and the FREAK algorithm for feature description, the initial matching quality is improved while maintaining a balance in speed. In the matching purification stage, a Hamming distance threshold is used for coarse filtering of match pairs, and the Hamming distances are sorted in ascending order to obtain a sequence of feature point matches from high to low similarity. This allows sequential sampling in the iterative computation of RANSAC. This approach not only compensates for the potential premature termination of iterations when using only sequential-sampling RANSAC (which might fail to yield the optimal homography matrix), but also makes up for the drawback of traditional Hamming distance filtering (which cannot guarantee a sufficient number of matches), and improves the computational efficiency of traditional RANSAC iterations.

The specific steps of ORB combined with FREAK for feature point extraction and description are as follows:

**Step 1.** For the initial matching pairs, remove those whose Hamming distance is below the distance threshold. Sort the Hamming distances in ascending order (i.e., descending similarity). From this ordered sequence, sequentially select four non-collinear feature point pairs, and re-estimate the initial homography matrix *H*.

**Step 2.** Use *H* to test the remaining matching pairs. Compute the reprojection error of the matched points under the current homography matrix. If the error of a pair is below the threshold, add it to the current inlier set.

**Step 3.** Dynamically adjust the upper bound of the iteration count according to parameters such as confidence level and the current number of inliers, then return to **Step 1**.

**Step 4.** Repeat the operation *M* times to complete *M* iterations. For each iteration, record the number of inliers and the transformation matrix *H*, and update the set with the maximum number of inliers.

**Step 5.** The matching pairs with the largest number of inliers are taken as the purified feature point set, which constitutes the final feature matching result.

### 3.2. Optical Flow Tracking of the AR Target Region

By extracting and matching image feature points and computing the homography matrix, camera pose estimation can be performed. However, processing each frame of an input video sequentially using feature point detection and matching is computationally expensive. Adopting a visual-feature based tracking approach can avoid repeated detection of target features, thereby improving the speed of feature point detection and tracking, as well as the efficiency of camera pose estimation. Although optical flow can handle feature point tracking in simple scenes, it fails when scene changes become complex and cause some feature points to be lost—for example, when the target moves out of bounds or is completely occluded—because those lost points cannot be recovered, eventually leading to target loss. Moreover, existing AR 3D tracking registration methods based on optical flow estimate the camera pose for each frame by accumulating inter-frame motion. If an erroneous pose estimate occurs in a previous frame, this approach will accumulate errors in subsequent frames, causing the camera pose tracking accuracy to gradually degrade over long-term tracking, and may even prevent successful AR 3D virtual–real registration.

**Figure 4:**
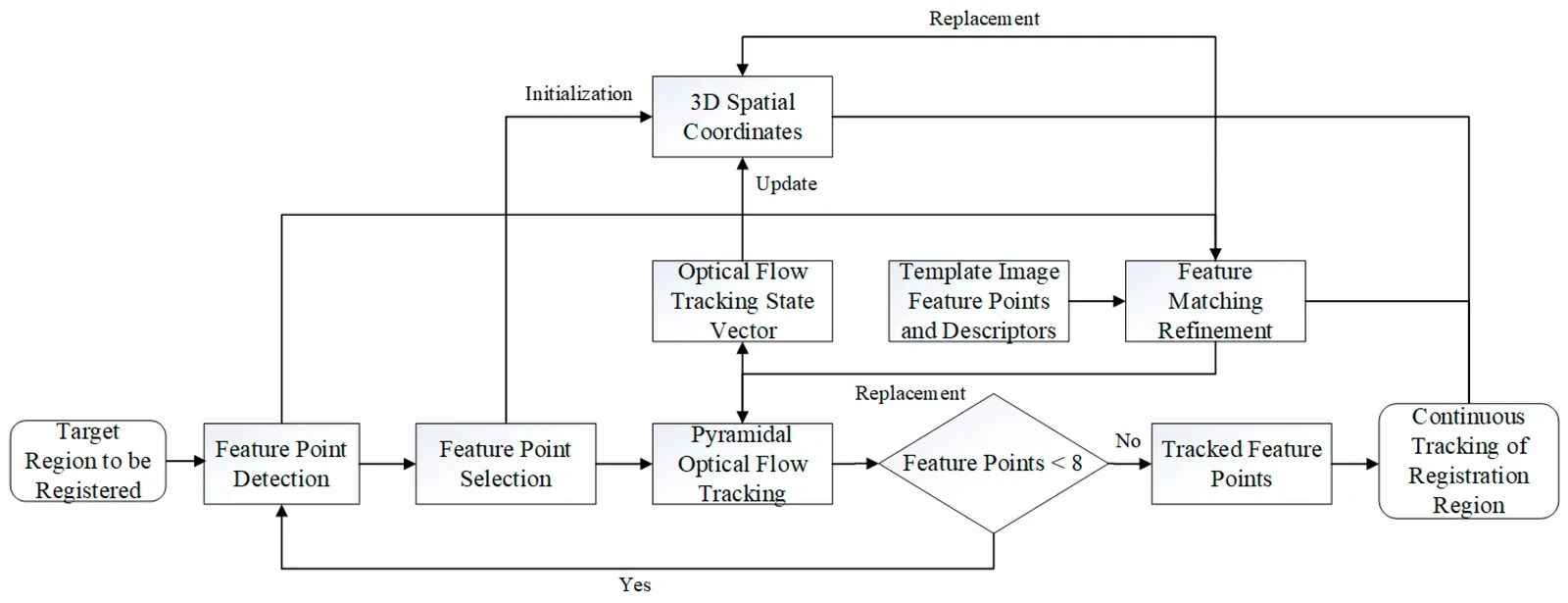
AR target area optical flow tracking.

As shown in Figure 4, before applying optical flow for target tracking, the feature points obtained from initialization detection are pre-processed. That is, feature points are filtered according to a distance threshold, retaining those that are uniformly distributed and have superior distinctiveness, to form the initial sequence for optical flow tracking. During the tracking and registration process, instead of accumulating pose changes between adjacent frames, the target in the first frame is retained as the template image. In subsequent changes, the spatial and planar coordinates are updated in real time based on the optical flow tracking results, and then the solvePnP function is used to robustly compute the camera pose. This approach better ensures the stability of pose tracking. As long as the number of tracked feature points remains above a certain level, even in cases of out-of-bounds or partial occlusion that cause feature loss, robust camera pose computation can still be performed. In cases of complete occlusion or even total loss, i.e., when the number of feature points falls below a certain threshold, the target template is matched with the current frame to replenish and update the optical flow tracking sequence, and the spatial coordinate points are replaced to immediately recover the camera pose. Through this method of feature point recovery and pose restoration, stable camera pose estimation and virtual model registration can be achieved during long-term tracking.

Currently, optical-flow-based tracking for camera pose estimation is solved by exploiting the relationship of feature points between adjacent frames. However, in practical applications, if the target to be registered moves out of bounds or some feature points are lost due to partial occlusion, the inter-frame errors accumulate. For long-term tracking and registration, the estimated pose becomes unstable and inaccurate. The optical flow method relies on the feature point sequence initialized at the start, and the feature points used for optical flow computation should have distinct characteristics so that the tracking is stable and reliable. Here, the ORB algorithm is adopted for corner detection, together with a distance constraint mechanism, which ensures that the distance between any two feature points is greater than a certain threshold. When the distance is smaller than this threshold, the points are considered to be clustered and are removed from the sequence. The final output is a set of uniformly distributed feature points after filtering, which improves feature quality and enhances processing speed.

To address the error-accumulation problem inherent in traditional optical-flow tracking, a direct matching scheme between 2D feature points and 3D spatial coordinate points can be used. That is, for all tracked feature points and their corresponding spatial coordinates (updated in real time), the PnP function is directly employed to solve for the camera extrinsic parameters. In addition, when traditional optical-flow computation is affected by occlusion or the target moving out of bounds, feature points may be lost; if no new feature points are extracted, the number of tracked points will gradually decrease, leading to tracking failure. Therefore, when the number of feature points falls below 8, an improved feature-point detection and matching approach is applied to the template image and the current frame (where points have been lost) to re-acquire a sufficient number of feature points for tracking, and the corresponding spatial coordinates are re-initialized, so that the camera pose computation can be resumed and the accuracy and stability of the optical-flow tracking are guaranteed.

In summary, to address the problems of existing optical-flow tracking—including feature point selection, target out-of-bounds, complete occlusion, and the consequent error accumulation in camera pose estimation due to feature loss—this paper proposes a feature-point selection scheme based on optical flow and a tracking-registration method with feature-point recovery. The steps of AR target-region optical-flow tracking are as follows:

**Step 1.** Initialize by selecting the target region, save the local area as a template image, and use the ORB algorithm for feature point detection; compute and save the feature points and their descriptors.

**Step 2.** Apply the distance constraint mechanism to filter feature points with good characteristics, and record the feature point coordinates as *Points_0*.

**Step 3.** Based on the detected 2D feature points *Points_0*, compute and record the corresponding 3D feature point coordinates as *Points3D*.

**Step 4.** Use the pyramid optical flow method to track the feature points in *Points_0*, compute their new position coordinates in the next frame, and save the indices of lost points as *MissPointsIndex*. If the number of lost points is greater than 0, update *Points3D*.

**Step 5.** Input the pre-computed *Points3D* and the corresponding tracked feature points *Points_0*, and use the PnP function to estimate the current camera pose, i.e., the rotation and translation matrices.

**Step 6.** Based on the computed rotation and translation matrices R,t and the internal parameters of the AR camera, use the OpenGL thread to render and draw the virtual model onto the real-scene image; simultaneously read the next frame and return to **Step 4** to repeat the subsequent operations.

**Step 7.** Judge the number of tracked feature points: if it is less than 8, the feature points are considered completely lost. In that case, perform feature-point detection and matching again between the target template and the current frame, and use the improved RANSAC algorithm for match purification.

**Step 8.** Using the method in **Step 7**, compute the target feature points in the current frame, re-update the feature point sequence *Points_0*, compute the corresponding 3D spatial coordinates *Points*_*3D*, and replace *Points3D* with these new values. Then go to **Step 4** and continue with the subsequent operations.

### 3.3. AR Target-Region Mean-Shift Tracking and Registration

**Figure 5:**
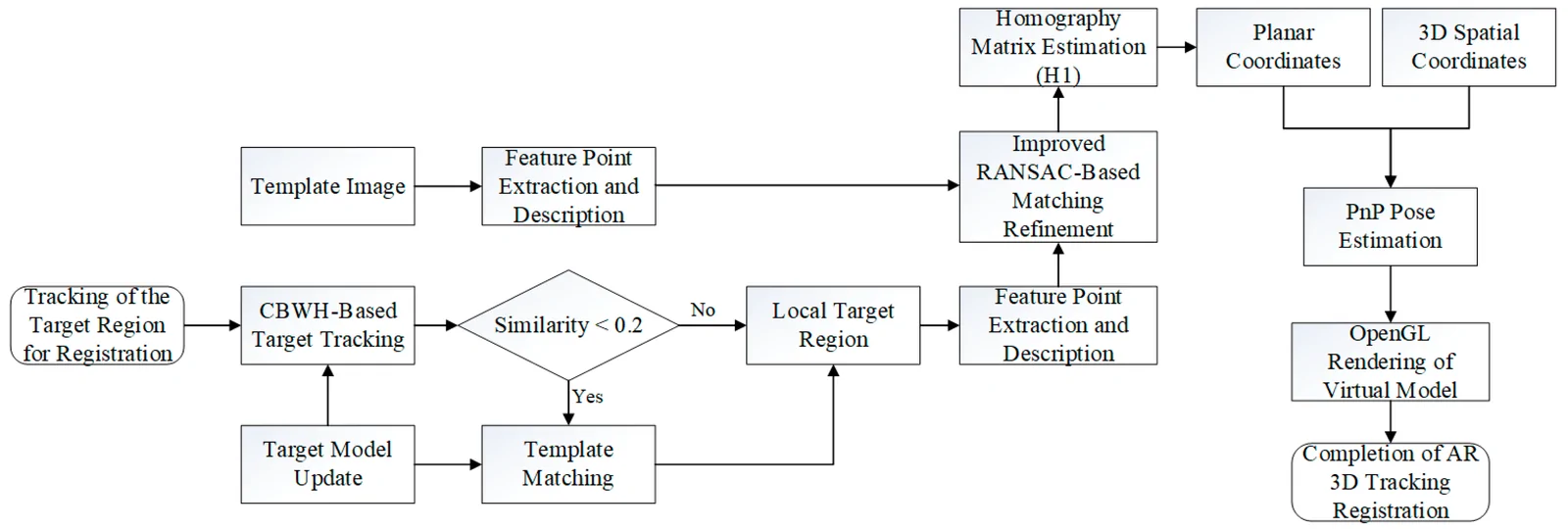
AR target area mean shift tracking registration.

Current mainstream AR 3D tracking-registration methods require global feature detection and matching on each frame, which is computationally expensive. Moreover, the feature points of the target to be registered are often not sufficiently rich, and they are susceptible to interference from non-target regions, resulting in poor matching quality and consequently affecting the accuracy of camera pose estimation. They are also subject to temporal and environmental constraints. By adopting a region-based tracking algorithm to assist the computation of camera pose via feature points, and by tracking the target region, the feature-detection area can be reduced and background interference suppressed. Then, an improved RANSAC algorithm is used for match purification and to estimate the optimal homography matrix, thereby improving the accuracy of feature matching and achieving real-time and accurate camera pose estimation. As shown in Figure 5, the CBWH tracking algorithm is relatively robust in complex backgrounds; however, it still suffers from the problem of long-term tracking loss under complete occlusion and similar conditions, and such target loss significantly impacts the AR virtual–real fusion effect. Therefore, based on the CBWH tracking algorithm, this paper introduces a template relocation strategy. When target loss is determined, a template-matching method is used to re-search for the target to ensure continuous and stable tracking. At the same time, the target template is updated in a timely manner when certain conditions are met, so as to achieve continuous and stable tracking of the target.

If the similarity of the target model in the current frame is less than 0.2, the target is considered lost, and a template matching method is used for relocalization. Template matching can detect the target region with the highest similarity in the image. By traversing the entire frame with the template image, the matching quality is evaluated by computing the sum of squared differences between the image and the template pixels. The best match corresponds to a value of zero, then:

(6) $$R\left(x,y\right)={\sum_{x^{\prime },y^{\prime }}\left(T\left(x^{\prime },y^{\prime }\right)-I\left(x+x^{\prime },y+y^{\prime }\right)\right)}^{2}$$

Among them, $\left(x,y\right)$ is the pixel value corresponding to any point in the template, and $\left(x+x^{\prime },y+y^{\prime }\right)$ is the pixel value of the corresponding overlapping point in the image. The region where the minimum value is obtained is the optimal matching result. However, template matching calculates the similarity between the target and the template and depends on whether the template is chosen appropriately. When the target undergoes affine transformation, the template matching performance is not ideal, making timely and accurate template updates very important. The similarity between the original background model and the current background model is measured by the Bhattacharyya coefficient:

(7) $$\rho =\sum_{u=1}^{m}\sqrt{{\hat{o}}_{u}{\hat{o}}_{u}^{\prime }}$$

If the similarity is less than 0.5, it indicates a significant change in the background, prompting an update of the background weight coefficients and the model, and the features of the initial target model are re-selected and recalculated. The similarity between the target model and the candidate target model is measured by the Bhattacharyya coefficient:

(8) $$\rho^{\prime }\left(y\right)=\sum_{u^{\prime }=1}^{m\prime }\sqrt{{\hat{p}}_{u}\left(y\right){\hat{q}}_{u}}$$

If the similarity is greater than 0.7, the target template used for template matching is updated to ensure matching accuracy. Therefore, a template relocalization strategy is proposed to address the target loss problem, thereby ensuring continuous and stable region-based tracking. In this paper, the mean-shift target locking module uses three fixed similarity thresholds: 0.2, 0.5, and 0.7, which are respectively used for preliminary screening of candidate regions, confidence discrimination of the target region, and confirmation of high-confidence regions. This distinguishes the foreground target from dynamic background interference based on feature descriptor matching similarity. Notably, 0.2 is a coarse screening threshold, filtering out candidate sub-regions with extremely low similarity to quickly exclude obviously irrelevant background regions; 0.5 is an intermediate discrimination threshold, used to distinguish candidate regions awaiting confirmation, falling between coarse screening and high confidence; 0.7 is a high-confidence confirmation threshold. When the region similarity exceeds this value, it is determined to be a reliable target region and is directly locked as the tracking window. The specific steps of the AR target-region mean-shift tracking and registration are as follows:

**Step 1.** Initialize by selecting the target region, save it as a template image, compute the background model and the target model, use the ORB combined with FREAK method for feature point extraction and descriptor generation, save and record the feature point coordinates *Points_0* and the feature descriptors, and define four planar coordinate points *corner0* and spatial coordinate points *Points3D*.

**Step 2.** Use the CBWH algorithm for target tracking to find the target position in the next frame, and update the target model in a timely manner according to the degree of change in the background model.

**Step 3.** Perform feature point detection on the searched target region, match these with the template image, and record the purified matching feature point coordinates as *Points_0* and *Points_1*.

**Step 4.** Use the improved RANSAC algorithm to estimate the homography matrix *H1* from *Points_0* and *Points_1*, and compute the four planar vertex coordinates corner1 of the current frame based on the perspective transformation of *H1*.

**Step 5.** Input the pre-computed *Points3D* and the corresponding feature points *Points_0* obtained in the current frame, and use the PnP function to estimate the current camera pose, i.e., the rotation and translation matrices.

**Step 6.** Based on the computed rotation and translation matrices and the internal parameters of the AR camera, use the OpenGL thread to render and draw the virtual model onto the real-scene image; simultaneously read the next frame, return to **Step 2**, and repeat the subsequent operations.

**Step 7.** If the similarity between the target model and the candidate target model is less than 0.2, the target is lost. Template matching is used to re-search for the target, the background model and target model are updated, and the process returns to **Step 3**.

### 3.4. Camera Pose Estimation for AR 3D Tracking Registration

The essence of AR 3D tracking registration is to achieve alignment between the real camera and the virtual information camera. The coordinate systems involved in AR 3D tracking registration include the object local coordinate system, the world coordinate system, the camera coordinate system, and the screen coordinate system. The camera pose is computed through the transformation relationships among these four coordinate systems, and based on this, the position and orientation of the virtual model are derived and rendered in the real scene, completing the AR 3D tracking registration.

In this paper, the target local rigid-body coordinate system and the standard pinhole camera coordinate system are defined. Using the pre-known physical dimensions of the target and based on the assumption of target planarity during initialization, fixed 3D reference points in the target local coordinate system are generated from the 2D features of the template image. During normal tracking, the 3D reference coordinates remain fixed, and only the 6DoF pose of the camera relative to the target is solved via PnP. When template relocalization is triggered to resume tracking, the algorithm does not recompute or update the 3D feature coordinates of the target. Instead, it directly reuses the static 3D reference points obtained during initialization and re-solves the camera extrinsic parameters using the 2D–3D match pairs output by relocalization. The target is treated as a rigid body, where the 3D feature coordinates in its local coordinate system are a priori constants; only the pose of the camera relative to the target changes.

**Figure 6:**
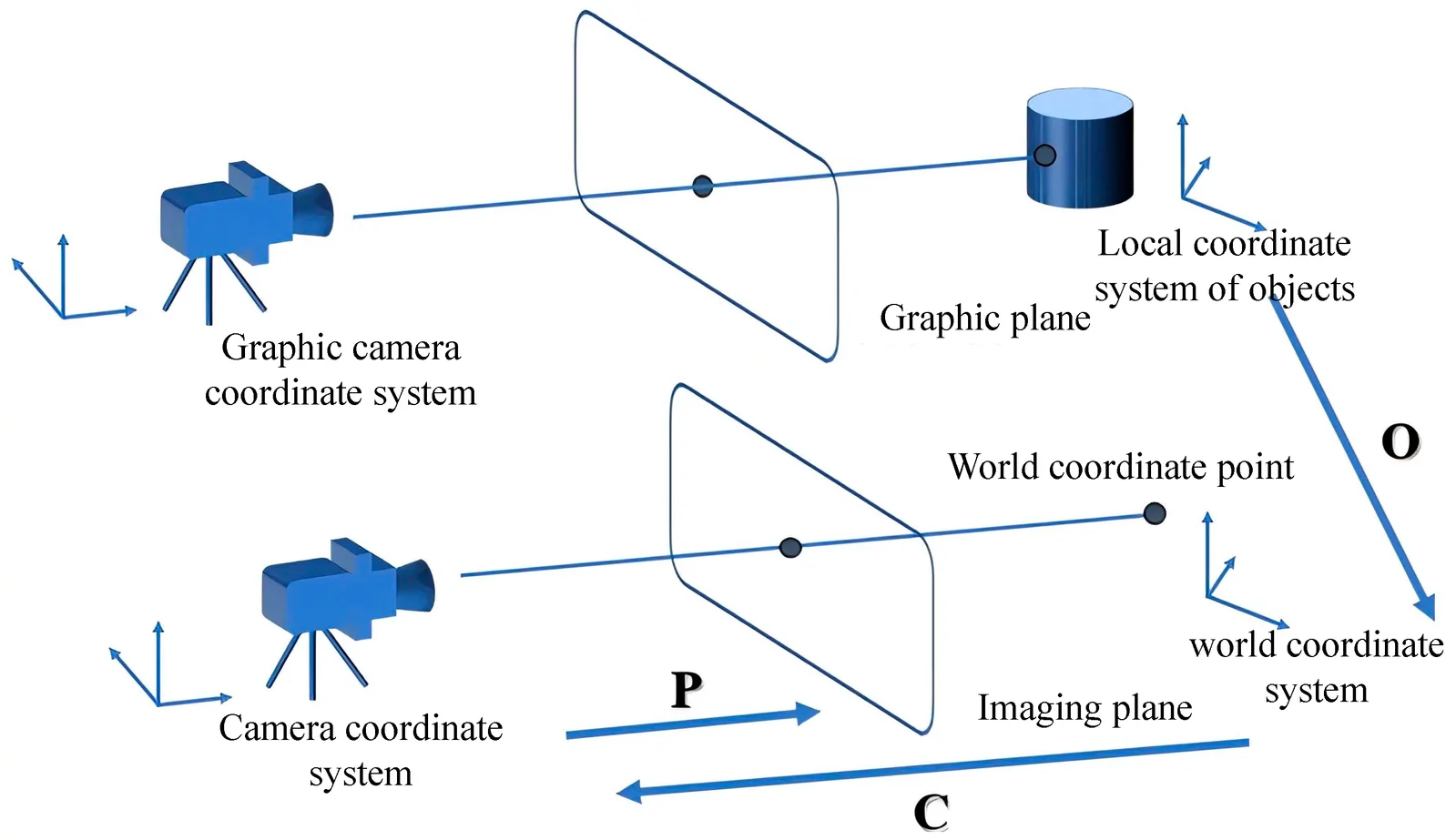
Transformation relationships among four coordinate systems.

The relationships among the four coordinate systems are shown in Figure 6. Among the three transformations between these coordinate systems, transformation C determines the extrinsic parameters of the camera, and transformation P determines the intrinsic parameters of the camera, which can be obtained through camera calibration. Specifically:

**(1) Transformation O:** the transformation from the virtual object coordinate system to the world coordinate system, which determines the position information for rendering and registering the virtual object in the real environment.

**(2) Transformation C:** the transformation between the world coordinate system and the AR camera coordinate system. This transformation mainly consists of two components: rotation and translation.

**(3) Transformation P:** the transformation from the AR camera coordinate system to the screen coordinate system, which maps and projects the 3D coordinates of the AR camera coordinate system onto the 2D coordinates of the screen.

The essence of AR 3D tracking registration is to solve in real time the position and orientation of the camera relative to the real object in the real world, which mainly includes camera intrinsic parameter calibration and extrinsic parameter solving. The computation of intrinsic parameters accomplishes the transformation from the camera coordinate system to the image coordinate system, and can be performed through pre-calibration. Tracking-registration methods based on visual features typically estimate the camera pose using the homography matrix obtained from feature-point matching, and require the use of the homography matrix to solve for the camera extrinsic parameters, i.e., the rotation and translation matrices. The camera extrinsic parameters implement the geometric transformation between the world coordinate system and the camera coordinate system, mapping a 3D point $\left(X_{w},Y_{w},Z_{w}\right)$ in the world coordinate system to the 2D point coordinate $\left(u,v\right)$ in the image coordinate system. From homogeneous coordinates can obtain:

(9) $$Z_{c}\left(\begin{array}{l} u\\ v\\ 1 \end{array}\right)=\left(\begin{array}{ccc} \frac{1}{dx} & 0 & u_{0}\\ 0 & \frac{1}{dy} & v_{0}\\ 0 & 0 & 1 \end{array}\right)\left(\begin{array}{ccc} f & 0 & 0\\ 0 & f & 0\\ 0 & 0 & 1 \end{array}\right)\left(\begin{array}{cc} R & t\\ 0^{T} & 1 \end{array}\right)\left(\begin{array}{l} X_{w}\\ Y_{w}\\ Z_{w}\\ 1 \end{array}\right)=\left(\begin{array}{ccc} f_{x} & 0 & u_{0}\\ 0 & f_{y} & v_{0}\\ 0 & 0 & 1 \end{array}\right)\left(\begin{array}{cc} R & t\\ 0^{T} & 1 \end{array}\right)\left(\begin{array}{l} X_{w}\\ Y_{w}\\ Z_{w}\\ 1 \end{array}\right)$$

(10) $$K=\left(\begin{array}{ccc} f_{x} & 0 & u_{0}\\ 0 & f_{y} & v_{0}\\ 0 & 0 & 1 \end{array}\right),M=\left(\begin{array}{cc} R & t\\ 0^{T} & 1 \end{array}\right)$$

**Figure 7:**
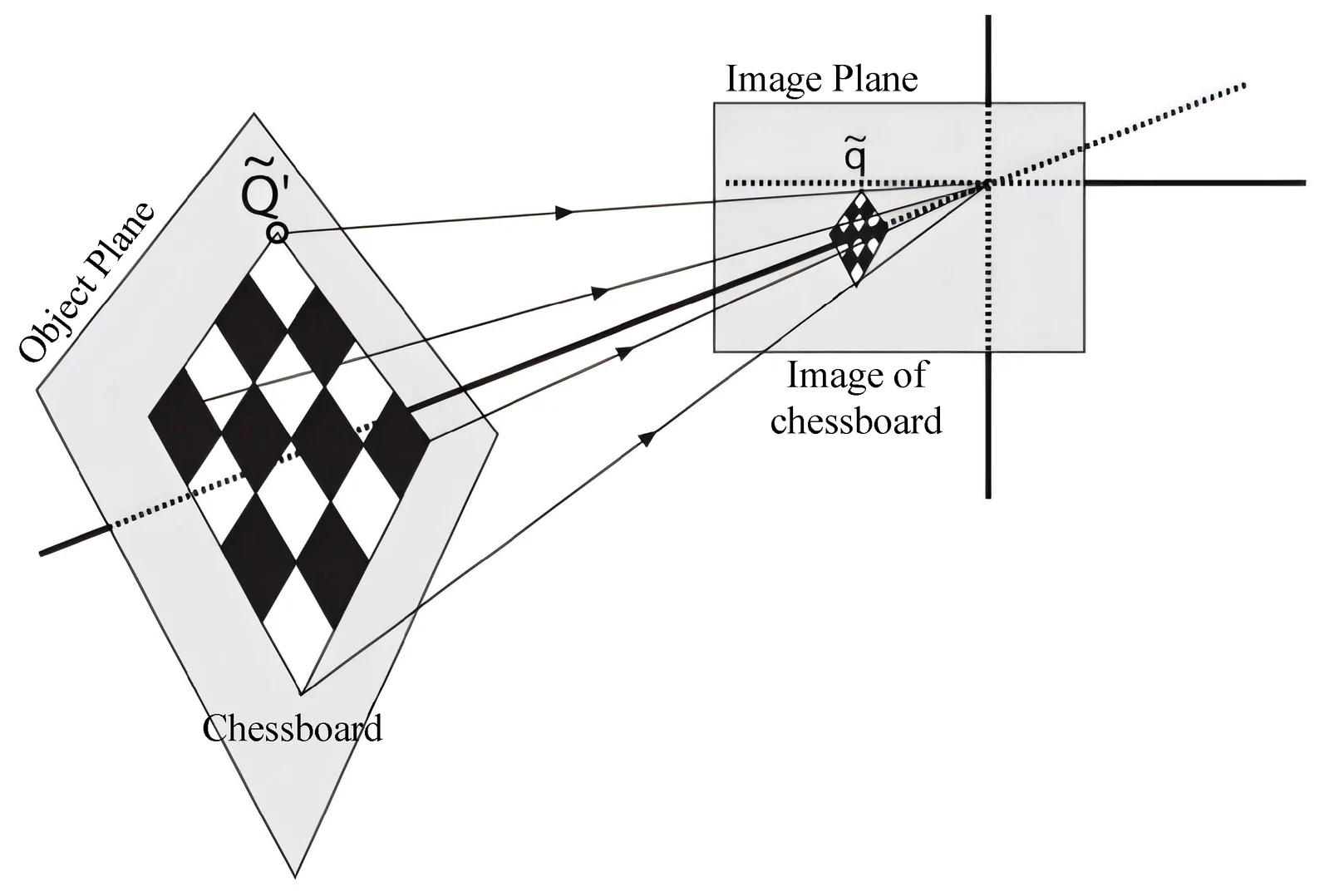
Mapping from object plane to image plane.

Among them, *K* is the camera intrinsic parameter matrix, and *M* contains the transformation relationship between the world coordinate system and the camera coordinate system in terms of rotation and translation components; that is, *M* is the camera extrinsic parameter matrix. The homography matrix reflects the perspective relationship between two images and can characterize the transformation of the camera pose. According to the camera imaging principle, the perspective mapping of the target on the image plane can be described by the homography matrix. As shown in Figure 7, a corresponding mapping relationship can be established between the homography matrix and the rotation-translation matrix R,t. Specifically, by matching feature points of the target object in the image plane and solving for the homography transformation, and then using the transformed feature points together with the known 3D spatial coordinates, OpenCV provides an interface for the PnP algorithm. This algorithm employs an iterative method based on Levenberg–Marquardt optimization to compute the optimal pose that minimizes the reprojection error. Finally, the resulting matrix R,t, which is the camera extrinsic parameter matrix is obtained, and completing the AR 3D tracking registration.

## 4. Experimental Results and Analysis

To comprehensively verify the overall performance of the lightweight monocular AR 3D tracking and registration method proposed in this paper, and to clarify the effectiveness of each algorithm module and its parameter adaptation rules, this study conducts comparative experiments with current mainstream feature matching, optical flow tracking, and 3D tracking-registration methods. Both qualitative and quantitative analyses are performed across multiple dimensions, including computational efficiency, tracking accuracy, and scene robustness.

The experiments are carried out on an HP graphics workstation with the following hardware configuration: Intel(R) Xeon(R) Gold 6330 2.00 GHz processor, NVIDIA RTX 4070 (16 GB VRAM) graphics card, and 64 GB system memory. The software platform is Ubuntu 20.04 LTS, with algorithm implementation and performance testing based on OpenCV 4.8.0, and virtual–real scene rendering and visualization using OpenGL 4.6. During the experiments, all algorithm parameters are fixed uniformly: the maximum number of ORB feature points per frame is set to 800, the FAST corner detection threshold is 20, the image pyramid has eight levels, and the scale factor is 1.2. The FREAK descriptor uses the standard 512-bit full dimension without dimensionality reduction. In the feature matching stage, the Hamming distance is used as the similarity metric, with bidirectional cross-checking enabled to filter effective feature pairs. A Lowe ratio test is also introduced to refine the matching results, with the ratio threshold set to 0.75 to precisely eliminate ambiguous false matches, ensuring the stability and accuracy of basic feature matching.

In addition, to further investigate the performance gains and parameter sensitivity of key mechanisms, ablation experiments are conducted on the feature pre-screening strategy and the tracking discrimination threshold. In the tests of FREAK 128-bit feature pre-screening, approximately 82% of candidate match pairs are filtered out, reducing the average per-frame matching time by 21%, which significantly improves algorithm efficiency. However, this also removes a small number of correct matches, causing a slight decrease in the success rate of tracking recovery. Meanwhile, the tracking discrimination threshold is traversed across the range of 5 to 12, and the relocation trigger frequency, false-trigger rate, missed-trigger rate, tracking recovery success rate, and per-frame computational cost are statistically recorded under different thresholds.

### 4.1. AR Camera Parameter Solving and Calibration

Camera intrinsic parameters and distortion coefficients are fundamental parameters that determine the accuracy of AR virtual imaging and ensure the consistency of virtual–real registration. AR 3D tracking registration solves the camera extrinsic parameters, i.e., the rotation and translation matrices, through the homography matrix between images. By combining these extrinsic parameters with the calibrated intrinsic parameters, the spatial position and orientation correspondences between the real-scene target and the virtual model can be determined, thereby achieving accurate overlay rendering of the virtual model. In actual imaging scenarios, the AR camera cannot satisfy the ideal pinhole imaging model, and nonlinear lens distortion is ubiquitous, which directly causes feature offsets, pose estimation deviations, and virtual–real fusion misalignment. Since the intrinsic parameters and distortion coefficients do not change with the field of view, they can be accurately solved through camera calibration, along with image distortion correction.

**Figure 8:**
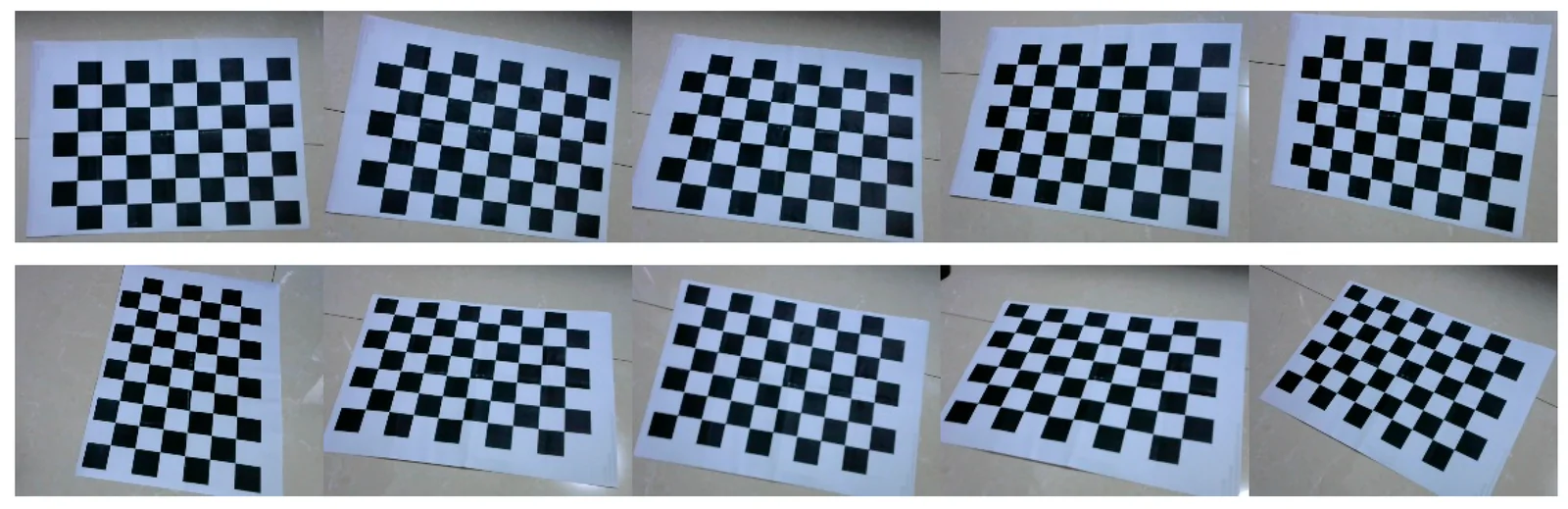
Checkerboard calibration image.

In this paper, the classic Zhang Zhengyou planar calibration method [31] is adopted for camera calibration, which effectively avoids the reliance of traditional calibration algorithms on high-precision custom calibration equipment. This method only requires capturing chessboard calibration images at different poses and viewing angles, and iteratively solves for the optimal camera intrinsic parameters, extrinsic parameters, and distortion coefficients through the homography transformation relationships of the images. It offers the advantages of simple operation, high calibration accuracy, and strong robustness. As shown in Figure 8, A Logitech C270 camera is used, with the image acquisition resolution uniformly set to 640 × 480. Ten sets of chessboard calibration images at different poses are captured from multiple angles for camera parameter solving and distortion correction. The intrinsic and distortion parameters obtained from this calibration are shown in Table 1 and Table 2, respectively. The calibration accuracy verification results show that the average reprojection error of the algorithm is 0.31 pixels, and the maximum reprojection error is 0.78 pixels. The overall error is within an acceptable range and can meet the requirements of high-precision AR tracking and registration.

To eliminate the interference of lens distortion on feature extraction, optical flow tracking, and pose solving, a pre-distortion-correction step is added to the algorithm pipeline. Before performing feature extraction, LK optical flow tracking, feature matching, and homography matrix solving, all real-time input images are undistorted using the calibrated intrinsic parameters and distortion coefficients. At the same time, the reference template images used in the tracking relocalization module are all taken from the undistorted standard images, ensuring that the imaging models of the reference image and the real-time frames are perfectly consistent. This avoids pose errors caused by distortion or inconsistent image formats, thereby guaranteeing the accuracy and stability of the subsequent AR 3D tracking and registration.

### 4.2. AR Feature Detection and Matching Experiment

**Figure 9:**
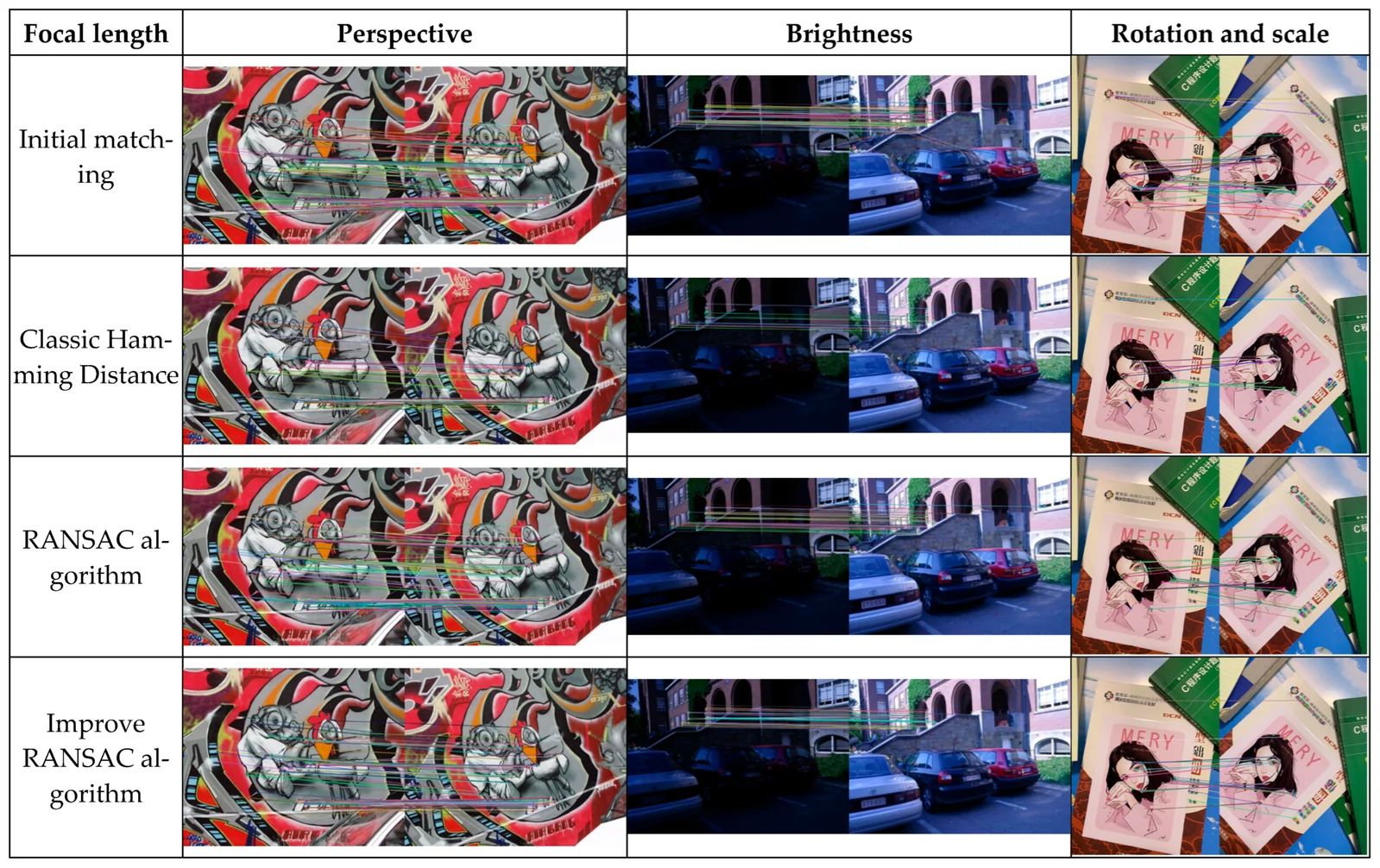
Feature detection and matching experiment.

**Figure 10:**
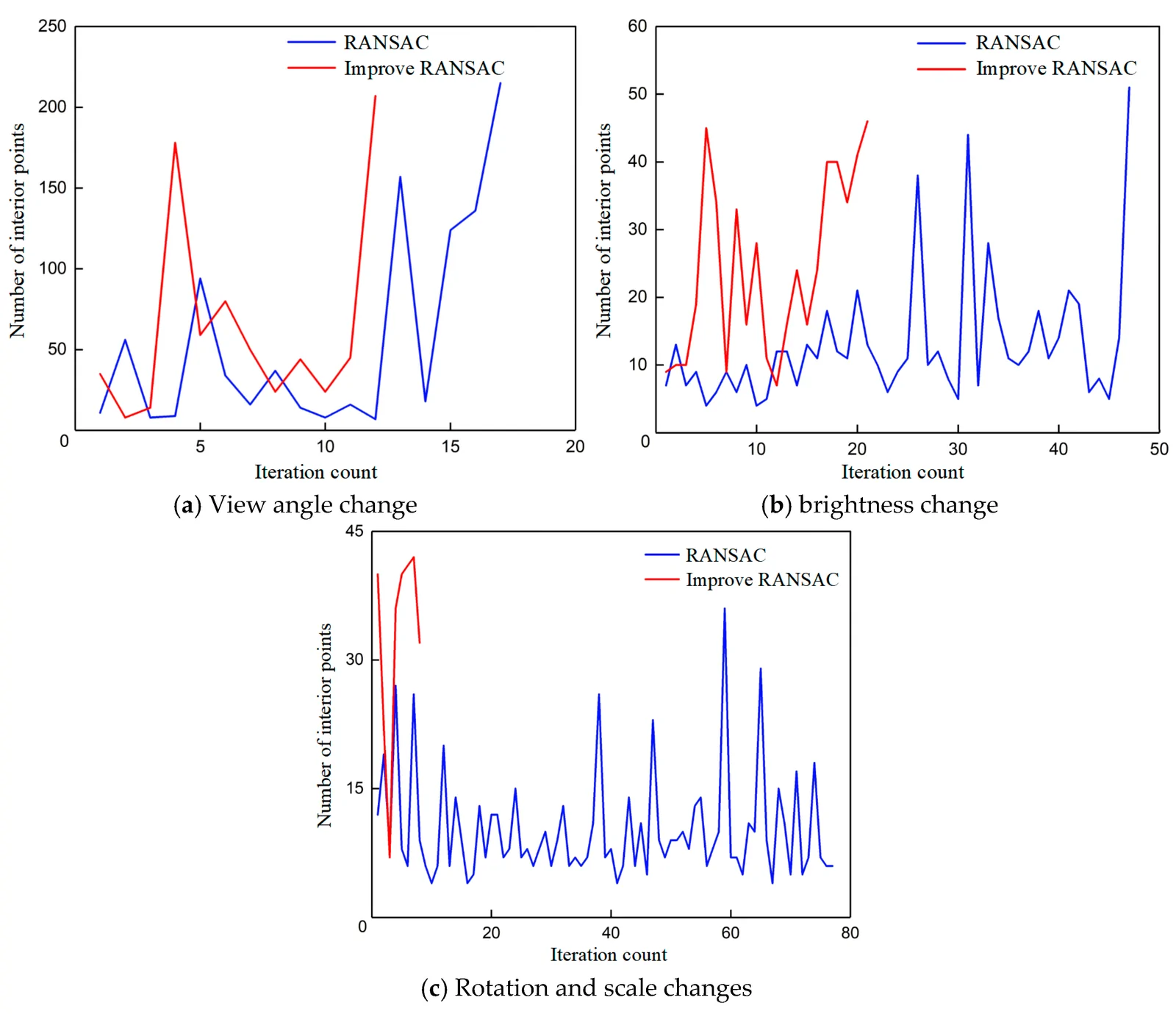
Changes in the number of interior points with iteration times under different variations.

To verify the effectiveness of the feature extraction method proposed in this paper, images from the Oxford dataset containing viewpoint changes and illumination variations are selected, together with real-scene images with rotation and scale changes. Comparative experiments are conducted between the classical Hamming distance approach, the traditional RANSAC algorithm, and the proposed method. The experimental results of image matching under viewpoint changes, illumination changes, and rotation and scale changes are shown in Figure 9. The quantitative experimental results are summarized in Table 3, Table 4 and Table 5. Figure 10 presents the comparison of the number of iterations and the number of inliers between the traditional RANSAC algorithm and the proposed method under the three types of image changes.

As shown in Figure 9, all three methods achieve relatively good results. However, from Table 3, Table 4 and Table 5, it can be seen that the proposed method outperforms the other two in terms of both higher accuracy and faster running speed. The classical Hamming distance method is very fast in processing, but it yields the lowest accuracy and the smallest number of final matched point pairs in the second set of experiments, the number of purified matches is less than half of that obtained by the other methods. Consequently, the empirical-threshold-based approach for filtering out false matches cannot guarantee a sufficient number of correct matches in certain application scenarios.

As shown in Figure 10, compared with the RANSAC algorithm, the proposed method significantly reduces the number of iterations, especially in the third set of experiments where match purification is completed in only eight iterations, effectively improving efficiency. In this paper, Hamming distance is used for coarse filtering without additional similarity computation, which efficiently increases the inlier ratio. Then, the Hamming distances are sorted in ascending order, and the homography matrix is estimated by sequential sampling. This approach not only ensures the quality of matching pairs but also improves the computational efficiency of the RANSAC algorithm. As a result, correct matching pairs can be obtained both quickly and accurately, and the optimal homography matrix can be estimated.

To further analyze the spatial-distance constraint on feature points, the threshold selection, and its sensitivity, and to evaluate the impact of the threshold parameter $d_{th}$ on system performance, a threshold-sensitivity experiment is conducted with candidate thresholds of {0, 8, 16, 24, 32}. The evaluation metrics include the coverage rate of feature points in the target region (%), the mean rotation error RE (°), the reprojection error TE (px), and the average per-frame processing time (ms). The experimental results are shown in Table 6. The feature-point coverage rate is defined as the percentage of retained features after filtering relative to the total number of originally detected features.

The experimental results show that when $d_{th}=0$, i.e., the distance constraint is disabled, the feature-point coverage rate is the highest, but the points are heavily clustered, which tends to increase the PnP solving error and the pose error. At the same time, the large number of features leads to higher computational overhead. As the threshold $d_{th}$ increases, the feature-point coverage rate decreases continuously, and the algorithm’s processing speed gradually improves. However, when the threshold is too large, the number of valid features becomes too small, the feature distribution in the target region becomes sparse, and the pose estimation error increases significantly. Balancing pose accuracy and processing speed, this paper selects $d_{th}=16$ px as the default threshold, under which the optimal overall performance is achieved. It should be noted that this threshold is defined in the image-pixel domain and is affected by image resolution; when the resolution changes, the minimum distance threshold should be re-scaled proportionally. Therefore, in complex environments with changes in rotation, scale, viewpoint, and illumination, the AR feature detection and match purification method proposed in this paper demonstrates good real-time performance and robustness.

### 4.3. AR Optical Flow Tracking Experiments and Analysis

**Figure 11:**
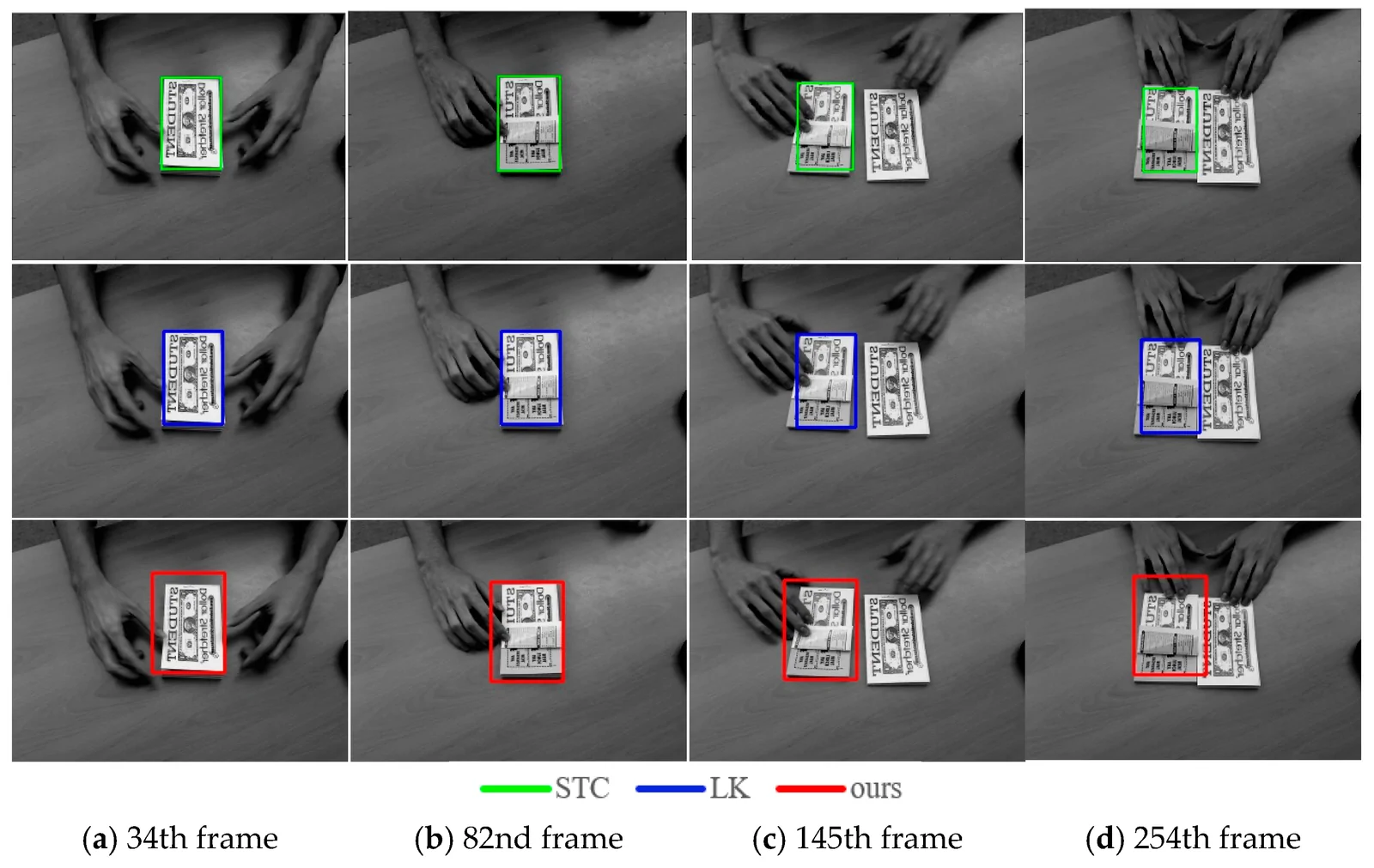
Comparison of tracking results for coupon sequence.

**Figure 12:**
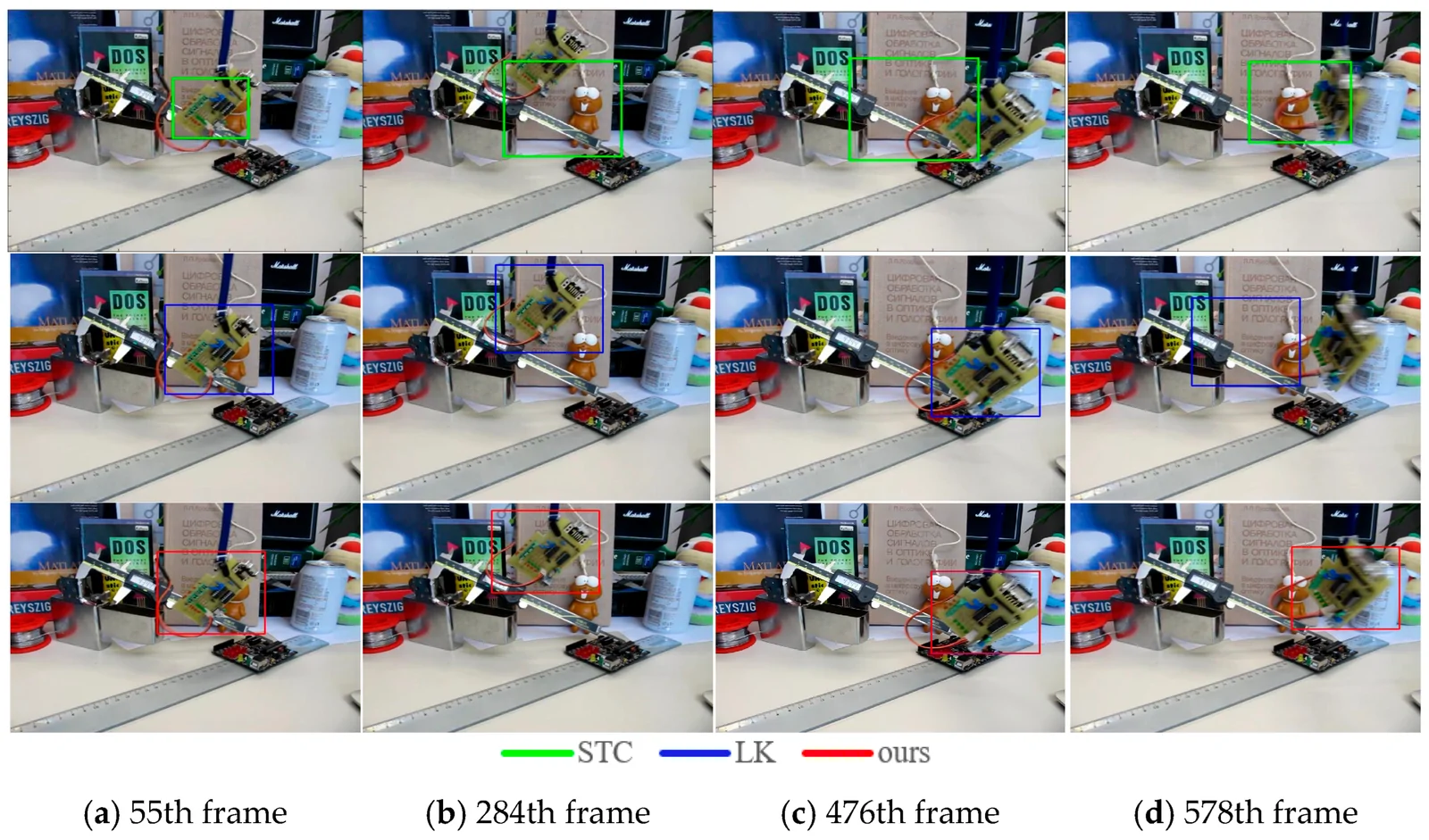
Comparison of tracking results for board sequence.

To verify the AR target tracking performance of the proposed method, experiments are conducted using two sequences from the public OTB dataset that contain background clutter, occlusion, and targets moving out of view. Although existing deep-learning-based AR 3D tracking-registration methods have achieved promising results, their complexity still makes it difficult to effectively match the hardware computational capabilities of mobile AR applications. For this reason, the proposed method is compared with the STC algorithm and the LK optical flow method, with all algorithm parameters tuned to their optimal values. To more intuitively illustrate the tracking accuracy of each algorithm, the center-error metric is used for quantitative analysis. The experimental results are shown in Figure 11 and Figure 12, from which it can be seen that the proposed method can effectively perform target tracking in all cases.

**Figure 13:**
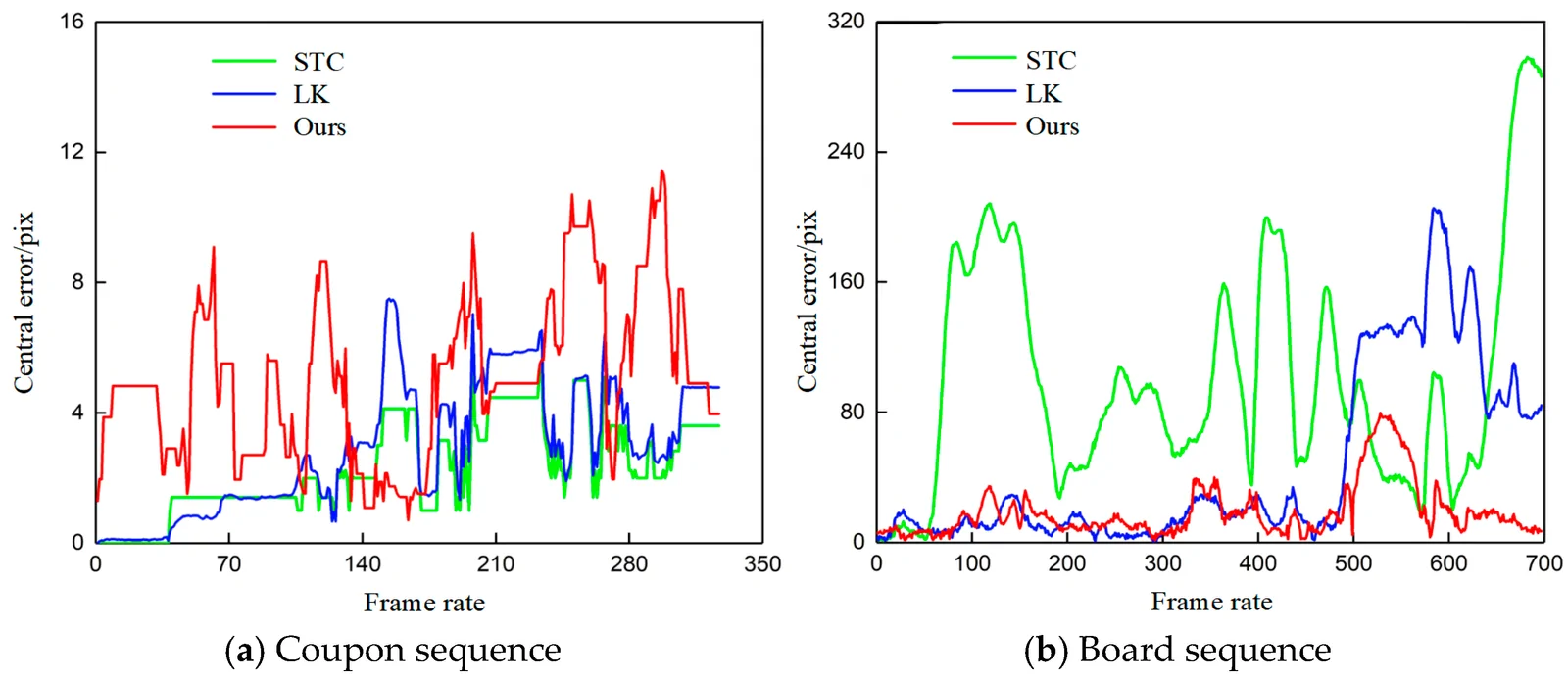
Comparison of center tracking errors under different sequences.

As shown in Figure 13, STC, LK, and the proposed method can all accurately track the target in the coupon sequence. However, as shown in Figure 13a, in terms of the center error, the two comparison methods are slightly superior to the proposed method. This is because the proposed method iteratively converges to the optimal position by computing the similarity between the target model and the candidate target model; especially under similar-background interference, the final iterative result exhibits minor fluctuations. Nevertheless, the maximum difference from the other two methods is only 4 px, which is still less than 12 px. As shown in Figure 13, STC already produces erroneous tracking after 50 frames and loses the target permanently; LK optical flow performs suboptimally, but it fails to track correctly after the target moves out of view at frame 487, and when the target reappears at frame 574 it cannot re-acquire the target. In contrast, the proposed method maintains good overall tracking performance. After the target reappears, template matching successfully relocalizes the target, and the method continues to track the target after frame 580, demonstrating good accuracy and stability.

In the AR optical-flow tracking pipeline, the number of valid inlier features after spatial-distance constraint and outlier removal is counted and used as the basis for judging the tracking status. When the number of remaining valid inlier matches within a frame falls below a threshold $N_{th}$, the current tracking is considered to have failed, and the template relocalization recovery procedure is triggered. The original implementation uses $N_{th}=8$ as the trigger threshold. To verify the rationality of this threshold, a multi-threshold sensitivity experiment is conducted, with candidate thresholds {4,6,8,10,12} selected for comparison. The evaluation metrics include the overall tracking success rate, the number of false-trigger relocalizations (recovery triggered incorrectly in normal tracking scenarios), the number of missed triggers (tracking has already drifted but recovery is not triggered), and the average processing time per frame. The experimental results are shown in Table 7.

The experimental results show that when the threshold $N_{th}=4,N_{th}=6$, a large number of frames that have already drifted in practice are not identified, the number of missed triggers increases significantly, many erroneous poses are output, and the overall tracking success rate decreases. Although the average processing time is relatively low, the tracking safety is poor. At $N_{th}=8$, a good balance is achieved between missed triggers and false triggers. This threshold can promptly identify tracking failures caused by occlusion and blur, while avoiding frequent false triggering of relocalization during normal tracking phases where feature counts experience only small temporary decreases. The overall tracking success rate reaches its highest value at this setting. When $N_{th}=10,N_{th}=12$, the threshold is further increased, and relocalization is triggered as soon as a small number of features are lost; the number of false triggers rises sharply. Even when tracking is still valid, a brief reduction in features repeatedly invokes template relocalization, introducing extra computational overhead and significantly increasing the processing time. Meanwhile, frequent relocalization introduces pose jumps, causing a decline in tracking success. Therefore, the threshold value has a substantial impact on the overall performance of the algorithm. When the threshold is too low, the system only initiates relocalization after severe feature degradation, which tends to cause persistent tracking drift. When the threshold is too high, a large number of ineffective false triggers occur, frequently invoking the template relocalization module, which significantly increases computational overhead and reduces real-time performance. A trade-off between accuracy and efficiency must be achieved through optimal threshold selection. The PnP pose solution requires at least four sets of 2D–3D correspondences, but four points can only provide a minimal solution and are extremely sensitive to noise and outliers, often yielding erroneous poses. Setting the threshold above the theoretical lower bound provides redundancy to accommodate noise and outliers. Based on the numerical stability of the PnP solver and the sensitivity experimental results, this paper selects $N_{th}=8$ as the tracking recovery trigger threshold.

### 4.4. AR 3D Tracking and Registration Experiments and Analysis

**Figure 14:**
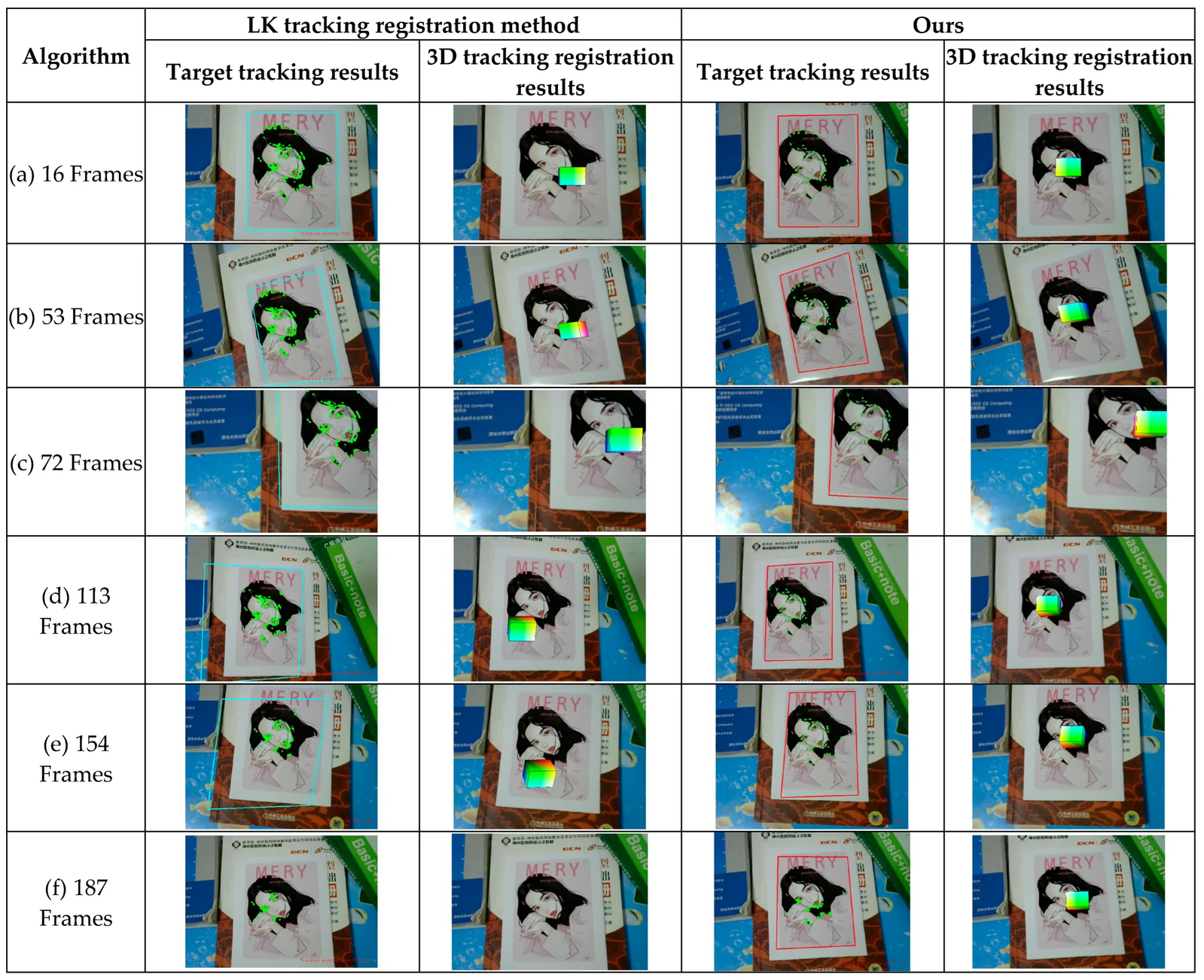
AR 3D tracking and registration experiment results.

To further analyze the performance of the proposed method in AR 3D tracking and registration, a comparison is made between the optical-flow-based tracking-registration method and the proposed method on real-scene AR 3D tracking-registration results. The green points depict the tracked feature points in the current frame, and the blue and red rectangles are redrawn based on the homography matrix, which can characterize the changes in the AR camera pose. The experimental results are shown in Figure 14.

As the frame number increases, the proposed method maintains a sufficient number of matched feature points under viewpoint changes, partial occlusion, and scale changes, and the camera pose represented by the red rectangle estimated from the homography matrix remains relatively accurate. In contrast, the homography matrix estimated by the optical-flow-based tracking-registration method is initially accurate; however, due to feature loss caused by the target moving out of bounds and occlusion, the accumulated motion error between adjacent frames causes the blue rectangle to change drastically and become deformed, as shown in Figure 14d,e. In Figure 14f, when the number of feature points decreases to a certain level, the method can no longer correctly compute the position and orientation of the target. Comparing Figure 14c–f, it can be seen that the proposed method directly matches the tracked 2D feature points with the corresponding 3D spatial coordinates (updated in real time) to compute the changing camera pose. The red rectangle remains accurate even in complex scenes and under target feature loss caused by out-of-bounds situations, intuitively demonstrating that the proposed method yields more accurate continuous pose estimation. Furthermore, when the target is completely lost and the number of feature points falls below 8, as shown in Figure 14f, the strategy of matching the template target with the current image is employed to obtain a new set of 2D feature points with sufficient quantity. The spatial coordinates are re-initialized, and the feature-point tracking sequence for optical flow is replenished and updated. The camera pose can still be correctly estimated, ensuring the stability of AR 3D tracking and registration during long-term tracking.

As shown in Figure 14, the color cube can still be accurately fused with the target region to be registered under camera motion changes. After partial occlusion, the proposed method introduces only minor interference to the virtual cube’s pose, and in subsequent changes it maintains the cube placed directly above the target without being affected. Compared with Figure 14c–f, as the number of frames increases, the pose of the virtual color cube rendered by the traditional method changes dramatically, with obvious shifts and rotations, until a large number of feature points are lost and AR 3D virtual–real tracking registration can no longer be properly performed. In contrast, with the proposed method, despite some influence from feature loss as the frame count increases, the virtual color cube can still be overlaid on the target object relatively accurately. As shown in Figure 14e, the virtual model’s pose undergoes only a minor change, and when the number of feature points decreases to a certain level, as shown in Figure 14f, the pose can still be recovered, ensuring correct overlay of the virtual model.

#### 4.4.1. Comparative Experiments and Real-Time Performance of AR 3D Tracking Registration

To comprehensively and objectively verify the performance of the monocular AR 3D tracking-registration method proposed in this paper, horizontal performance comparisons are conducted with traditional classical algorithms, recent mainstream registration methods, and state-of-the-art deep-learning-based SLAM algorithms. The experimental baseline algorithms include the classical LK optical flow and STC tracking algorithms. The mainstream deep-learning-based comparison methods encompass the lightweight local feature matching network LightGlue-ORB [32], SuperGlue-SIFT [33], the semi-dense matching algorithm E-LoFTR [34] designed for outdoor scenes, the end-to-end monocular 6DoF tracking model RGBTrack [35], the NeRF-template-assisted PixTrack tracking method [36], and the Improved-RBOT 3D tracking algorithm [37]. To further benchmark against the latest research in the field, this paper also introduces the lightweight deep SLAM algorithm Deep-SLAM [38] and the modular deep XRDSLAM tracking method [39] for quantitative accuracy comparison. The experiments evaluate overall performance from three dimensions: pose estimation accuracy, robustness in complex scenes, and real-time performance. The main evaluation metrics include the mean rotation error (RE), the reprojection error (TE), and the tracking success rate, where the tracking success rate is defined as the percentage of valid tracking frames in the test sequence. The experimental results are presented in Table 8.

The experimental results show that the traditional LK optical flow and STC algorithms exhibit large pose estimation errors and insufficient tracking stability in complex scenes such as illumination fluctuations and texture deficiency. Mainstream deep-learning methods such as SuperGlue-SIFT and PixTrack achieve excellent tracking accuracy due to their learned feature representation capabilities; however, they have large model sizes and complex inference pipelines, with per-frame processing times typically exceeding 30 ms on CPU, making it difficult to meet the real-time requirements of AR tracking and registration. E-LoFTR and LightGlue-ORB achieve a certain trade-off between accuracy and computational cost, but they still suffer from the inherent computational overhead of network inference and impose high demands on embedded deployment. In contrast, the proposed method is based on a feature-oriented architecture with no model parameters or CNN convolution overhead. It also optimizes the RANSAC iteration mechanism through Hamming distance pre-filtering of false matches, effectively reducing redundant computation in geometric solving and significantly improving overall algorithm efficiency. The SLAM comparison results indicate that Deep-SLAM improves matching stability in complex scenes through learned features, but its real-time performance is limited, while XRDSLAM combines multi-scale features with backend optimization and exhibits excellent robustness, yet its larger model size makes it difficult to adapt to low power devices. The overall computational pipeline of the proposed method is a linear operational architecture, mainly consisting of ORB-FREAK feature extraction, Hamming distance feature matching, optimized homography solving, optical-flow pose tracking, mean-shift target constraints, and template relocalization modules. Moreover, the Hamming distance coarse screening effectively removes invalid match pairs, compresses the RANSAC iterative sampling space, avoids the large computational cost and poor stability of traditional random-iteration algorithms, and further reduces overall computational complexity.

#### 4.4.2. Adaptability Analysis of AR 3D Tracking Registration in Dynamic Complex Environments

To further evaluate the algorithm’s adaptability to highly dynamic scenes, tests are conducted in complex environments such as urban outdoor and highly active indoor scenes, where there is substantial motion of foreground objects and continuous background disturbances. The test sequences include interference from moving pedestrians, moving objects, and continuously changing background content. The comparison methods are the same as those in Table 8, and the evaluation metrics remain rotation error, reprojection error, and tracking success rate. The experimental results in dynamic environments are shown in Table 9. The main disturbances in urban dynamic scenes are moving pedestrians and vehicles, while in highly active indoor scenes they are a large number of moving people and moving objects.

The experimental results show that in highly dynamic environments, moving objects in the background tend to introduce a large number of false matching points, causing drift in pose estimation. The traditional LK optical flow and STC algorithms have weak resistance to foreground motion interference; feature points are prone to drift in dynamic backgrounds, the pose error increases significantly, and the tracking success rate is relatively low. Deep feature-matching methods such as LightGlue-ORB, SuperGlue-SIFT, and E-LoFTR have stronger feature discrimination capabilities and can suppress some dynamic outliers, but when the proportion of moving objects is too high, they still generate a large number of false matches. Moreover, RGBTrack, PixTrack, and Improved-RBOT are easily disturbed by moving foreground objects, causing target drift. The Deep-SLAM method relies on backend graph optimization and outlier rejection, which provides a certain degree of robustness against dynamic interference; however, when dynamic objects occupy a high proportion, they can corrupt map construction and lead to tracking failure, while also incurring high computational overhead. In contrast, the proposed method employs a combination strategy of background weighted mean-shift target locking, Hamming distance threshold filtering, and region constrained screening of matching pairs, preferentially retaining target feature points and filtering out feature matches originating from moving foregrounds and dynamic backgrounds. This enables the method to resist interference from dynamic objects to a certain extent, maintaining the lowest pose error and the highest tracking success rate in moderately dynamic scenes such as urban outdoor and highly active indoor environments, and effectively adapting to scenarios with target motion and local dynamic disturbances in the surroundings.

#### 4.4.3. Comparative Experiments of AR 3D Tracking Registration in Complex Extreme Scenarios

To analyze the robustness of the proposed method in complex extreme scenarios, supplementary tests are conducted under three types of extreme conditions: insufficient lighting, rapid motion, and severe occlusion. The comparison methods are the same as those in Table 8, and the experimental results of each method under extreme scenarios are shown in Table 10.

The experimental results show that the traditional LK and STC algorithms exhibit the most significant performance degradation under the three types of extreme conditions. Under low light conditions, the image signal to noise ratio decreases, and fewer valid features can be extracted; rapid motion introduces motion blur and causes optical-flow drift; severe occlusion leads to extensive loss of target features, resulting in significantly increased errors and reduced tracking success rates. Deep-learning-based local feature methods such as LightGlue-ORB, SuperGlue-SIFT, and E-LoFTR are robust to illumination changes; however, under rapid motion blur and severe occlusion, the quality of feature description degrades, mismatches increase, and the network inference latency exacerbates the pose lag problem in dynamic scenes. RGBTrack, PixTrack, and Improved-RBOT, as end-to-end and template-based 3D tracking algorithms, perform reasonably well under partial occlusion, but their generalization capability is limited under low light and strong motion-blur conditions. Deep-SLAM, relying on learned features and backend optimization, achieves overall robustness superior to traditional methods and some deep-learning feature-matching methods; however, when strong motion blur or large-area occlusion occurs, it still suffers from feature failure and insufficient backend constraints. In contrast, the proposed method leverages a multi-module synergy of Hamming distance coarse filtering, regional distance constraints, optical-flow pose correction, background weighted mean shift, and template-matching relocalization. Under low light conditions, the ORB-FREAK illumination-robust descriptors are used to retain valid features and suppress mismatches. In high speed motion scenarios, real-time pose correction via optical-flow sequences mitigates drift caused by motion blur. When severe occlusion occurs, the template relocalization mechanism is triggered to maintain tracking continuity under local feature loss. As a result, the proposed method achieves the lowest rotation error, the lowest reprojection error, and the highest tracking success rate across all three extreme conditions.

#### 4.4.4. Performance Evaluation of AR 3D Tracking Registration on Different Hardware Platforms

To evaluate the cross-hardware portability of the algorithm, tests are conducted on three representative hardware platforms: PC, embedded edge board, and mobile phone. The input image resolution is uniformly set to 640 × 480, GPU/NPU hardware acceleration is disabled throughout, and all algorithm modules are covered in the tests. The evaluation metrics include average processing time per frame, average frame rate, and average CPU utilization. The test results are shown in Table 11.

The experimental results show that the PC side with sufficient computing power can stably achieve 51.3 fps, fully meeting the real-time requirements of AR tracking. On the Jetson Nano embedded platform, the frame rate reaches 27.2 fps under pure CPU operation, enabling near-real-time performance in complex scenes. On an ordinary Android mobile device, the average frame rate under CPU-only operation is 20.7 fps; although it can still perform tracking, the CPU utilization is as high as 84%, causing noticeable device heating. In complex scenarios with dense features and frequent triggering of template relocalization, the per-frame processing time on the mobile device further increases to 55–65 ms, and the frame rate drops to 15–18 fps, with obvious frame-rate jitter that cannot consistently meet real-time requirements. Compared with deep-learning-based methods, the proposed method has no network inference overhead and consumes less time on equivalent hardware. Therefore, the proposed method performs well in real time on PC and embedded platforms, and remains usable on mobile devices.

### 4.5. Ablation Experiments for AR 3D Tracking Registration

To quantitatively analyze the contribution of each module to the tracking-registration accuracy, robustness, and real-time performance, ablation experiments are conducted. The effects of four main modules are sequentially verified: (A) Hamming distance coarse filtering and ascending-order sampling for homography matrix solving; (B) distance constraint in the target registration region and optical-flow pose correction; (C) background weighted mean-shift reduction of the detection range; and (D) template-matching relocalization and dynamic model updating. The experimental results are shown in Table 12.

The experimental results show that the Baseline, which only uses ORB-FREAK basic feature detection and matching together with traditional RANSAC for homography solving and does not incorporate any of the improved strategies proposed in this paper, suffers from a large number of false matches, low efficiency in homography solving, large rotation and reprojection errors, a tracking success rate of only 63.7%, and a per-frame processing time of 27.6 ms. When only Module A is enabled, the Hamming distance threshold eliminates many false match pairs, and the ascending-order sequential sampling iteration strategy reduces the number of invalid iterations. The per-frame processing time drops to 22.3 ms, the pose estimation accuracy improves significantly, and the tracking success rate increases to 74.2%. When Module B is introduced on the basis of A, the distance constraint in the target registration region removes interfering feature points outside the region, and the feature matching is used to correct the optical-flow tracking sequence and camera pose in real time, alleviating pose accumulation errors. The rotation error decreases to 2.47°, and the tracking success rate further improves to 81.5%. After adding Module C (background weighted mean shift), feature detection is limited to the target tracking region, suppressing interference from irrelevant features in complex backgrounds and further reducing the computational cost of feature detection, with the per-frame processing time dropping to 20.1 ms. Finally, when all modules, including D, are enabled, the template-matching relocalization and dynamic model updating strategies are added. In cases of sudden brightness changes or viewpoint shifts that cause temporary feature loss, the system can recover tracking, boosting the success rate to 90.1%. The final complete algorithm achieves an average rotation error of 1.86°, a reprojection error of 3.14 px, and a per-frame processing time of 19.5 ms. The ablation results demonstrate that each improved module brings positive gains in terms of accuracy, robustness, or real-time performance. Module A mainly optimizes matching quality and matrix-solving efficiency; Module B suppresses cumulative pose drift; Module C reduces computational load by narrowing the detection range; and Module D significantly enhances the stability of sustained tracking under extreme disturbances. The modules work together, enabling the algorithm to adapt to complex AR 3D tracking-registration scenarios involving rotation, scale changes, viewpoint shifts, and illumination variations.

## 5. Discussion

Lightweight AR 3D tracking and registration technology based on visual features, owing to its low power consumption, high real-time performance, and ease of deployment, has always been a mainstream research direction for mobile and embedded AR scenarios. To address the common problems of existing visual tracking and registration methods such as insufficient feature matching accuracy, weak robustness in complex environments, long-term tracking error accumulation, and poor stability in dynamic scenes this paper constructs a lightweight AR 3D tracking and registration framework that integrates optical flow tracking and mean shift. The algorithm achieves performance optimization from three levels through multi-module collaborative optimization: low-level feature matching, mid-level pose tracking, and high-level region constraint. First, it fuses the ORB-FREAK feature operators to build an efficient feature extraction and matching system, improves the traditional RANSAC random sampling strategy, and optimizes the homography matrix solution by combining Hamming distance based mismatching filtering and an ordered iterative sampling mechanism. This enhances feature matching accuracy while controlling computational overhead, effectively adapting to complex scene variations such as scale scaling, rotation, viewpoint shifts, and illumination fluctuations. Second, it improves the traditional optical flow tracking mechanism by selecting high-confidence feature points through distance constraints within the target region. Relying on the 2D–3D feature mapping relationship, it achieves accurate pose estimation, significantly suppressing error accumulation in long-term tracking, and enables rapid pose recovery through dynamic feature re-matching to ensure tracking continuity. Finally, it introduces the CBWH (Centroid-based background weighted Histogram) mean shift region tracking strategy, combined with template relocalization and dynamic target model update mechanisms, effectively solving registration failures caused by target occlusion, temporary loss, and dynamic background interference. Experimental results show that the proposed method can balance real-time performance, accuracy, and anti-interference capability on low power devices, meeting the deployment requirements of lightweight AR applications in complex scenarios.

Compared with existing mainstream AR tracking and registration methods, our method effectively compensates for the difficulty of traditional feature matching algorithms in balancing matching accuracy and computational efficiency. In particular, it addresses the weak anti-interference capability and prominent long-term drift issues of classical optical flow tracking algorithms, as well as the fact that traditional mean shift algorithms only provide region tracking without precise pose estimation, making them prone to tracking loss and virtual–real misalignment under complex scenarios such as severe illumination changes, abrupt pose variations, and partial occlusion. Meanwhile, most existing improved methods focus only on optimizing a single module, lacking a systematic collaborative design of feature matching, target tracking, and pose estimation, resulting in limited overall generalization improvement. Although deep learning-based AR registration methods have significant accuracy advantages, their large inference overhead and high computing power dependency make them unsuitable for lightweight deployment on mobile and embedded edge devices. Different from the above single-optimization approaches, our method adopts a lightweight multi-module collaborative optimization strategy based on target features and geometric constraints. Without significantly increasing algorithmic complexity, it simultaneously improves matching efficiency, pose accuracy, anti-interference capability, and long-term stability, providing a reference for low-computing-power, high-reliability AR 3D tracking and registration.

Our method alleviates short-term error accumulation through 2D–3D feature correspondences, but it does not incorporate keyframe constraints or global graph optimization mechanisms. Consequently, minute errors in ultra-long-term and large-scene continuous tracking cannot be completely eliminated, making it difficult to support more complex immersive virtual–real interaction in 3D dynamic scenes [40]. Compared with state-of-the-art deep learning-based SLAM methods, our approach extracts fewer effective features in scenarios with sparse target textures, resulting in matching accuracy and pose stability inferior to learning-based deep feature algorithms [41]. Moreover, it relies solely on local template matching for relocalization and lacks global mapping and loop closure detection capabilities [42]. In view of these limitations, future work will focus on two directions: dimensional expansion and mechanism optimization. On one hand, we will integrate 3D model matching and depth sensing information to enhance the realism and alignment of virtual–real fusion in 3D scenes while preserving the lightweight advantages. On the other hand, we will introduce SLAM keyframe constraints and graph optimization theories, mining inter-frame motion correlations to build a global pose optimization model. Through global iterative correction, we aim to suppress error accumulation in ultra-long-term and large-scene tracking, further improving pose estimation accuracy and long-term stability [43]. Additionally, lightweight deep features can be incorporated to assist optimization, compensating for the shortcomings of handcrafted features in sparse-texture and extreme-interference scenarios, and enhancing global constraint capabilities. This will continuously improve the scene adaptability of the algorithm and promote the iterative upgrading of lightweight AR tracking and registration technology towards full-scene, high-precision, robust, and deployable solutions.

## 6. Conclusions

Visual 3D tracking and registration is the key to achieving AR virtual–real fusion and innovative applications. However, existing visual feature-based tracking and registration methods generally suffer from limited feature matching accuracy, low iterative computational efficiency, cumulative pose errors in long-term tracking, and insufficient robustness in complex scenarios, making it difficult to meet the high-precision real-time 3D registration requirements in dynamic and complex environments. To address these issues, this paper proposes an AR 3D tracking and registration method that integrates optical flow tracking and mean shift. First, we combine ORB and FREAK feature operators to build a high-performance feature detection and matching model, and introduce Hamming distance for coarse filtering of mismatched point pairs, effectively improving the inlier ratio and matching reliability. Then, a distance constraint strategy within the target region is adopted to enhance the quality of feature point selection. Relying on the precise correspondence between 2D image feature points and 3D spatial coordinates, we suppress cumulative camera pose errors, and employ a feature re-matching mechanism to rapidly recover lost tracking sequences and camera poses, ensuring tracking continuity. Finally, the CBWH mean shift algorithm is introduced to achieve stable tracking of the target region, combined with template-based relocalization and dynamic target model update strategies to enhance the algorithm’s environmental anti-interference capability. Meanwhile, an improved feature algorithm is used to solve the homography matrix with high precision, simultaneously improving both pose estimation accuracy and computational efficiency. Experimental results demonstrate that the proposed method can effectively handle complex scenarios such as target occlusion, transient target loss, and sudden illumination and pose changes, meeting the real-time requirements of AR systems. It effectively compensates for the technical shortcomings of traditional visual AR 3D tracking and registration methods, with significant overall performance improvement. In future work, we will introduce SLAM graph optimization techniques to refine inter-frame pose constraints, addressing the problems of motion error accumulation and drift in long-term tracking. Additionally, we will integrate 3D model geometric features and scene depth information into practical AR application scenarios, and conduct research on multi-target, lightweight 3D tracking and registration methods, further enhancing the realism and scene adaptability of virtual–real fusion, and promoting the engineering deployment and performance upgrading of AR 3D tracking and registration technology.

## Figures and Tables

**Table 1:** Internal parameters of AR camera measured in experiments.

Focal Length	Internal Parameters
Focal length *f_x_*	814.6903825704524
Focal length *f_y_*	815.7649243269345
Principal point offset *c_x_*	300.2247996135054
Principal point offset *c_y_*	218.361552246424

**Table 2:** Distortion parameters of AR camera measured in experiments.

Focal Length	Distortion Parameter
Radial distortion parameter *k*1	0.1377881797236274
Radial distortion parameter *k*2	−0.5945605988359182
Radial distortion parameter *k*3	−0.01476725249653015

**Table 3:** View angle change images with a size of 800 × 600.

Algorithm	Initial	Filter	Purification	Accuracy	Iteration Count	Time(s)
Classic Hamming Distance	271	-	80	29.52	-	0.0529813
RANSAC		-	219	80.8	17	0.115491
Improve RANSAC		234	215	91.88	12	0.0679303

**Table 4:** Brightness change image with a size of 921 × 614.

Algorithm	Initial	Filter	Purification	Accuracy	Iteration Count	Time(s)
Classic Hamming Distance	89	-	15	16.85	-	0.0206627
RANSAC		-	52	58.42	47	0.1290900
Improve RANSAC		70	48	68.57	21	0.0533624

**Table 5:** Rotation and scale change images with a size of 480 × 640.

Algorithm	Initial	Filter	Purification	Accuracy	Iteration Count	Time(s)
Classic Hamming Distance	71	-	24	33.8	-	0.028976
RANSAC		-	41	57.75	77	0.154738
Improve RANSAC		50	40	80	8	0.0373462

**Table 6:** Sensitivity analysis of the minimum spatial distance threshold.

Minimum Distance Threshold $d_{th}$ (px)	Feature Point Coverage Rate (%)	Rotation Error RE/(°)	Reprojection Error TE/(px)	Average Time per Frame(ms)
0 (no distance constraint)	92.7	2.84	4.71	24.2
8	81.3	2.31	3.88	21.4
16 (selected in this paper)	67.5	2.15	3.56	19.5
24	52.2	2.47	4.13	17.8
32	38.6	3.06	4.94	16.3

**Table 7:** Sensitivity analysis of the tracking recovery trigger threshold $N_{th}$.

Trigger Threshold $N_{th}$	Tracking Success Rate (%)	Number of False-Trigger Relocalizations	Number of Missed Triggers	Average Time per Frame (ms)
4	81.2	12	147	18.7
6	84.8	27	83	19.1
8 (selected in this paper)	86.7	41	36	19.5
10	83.5	96	18	21.2
12	78.1	164	7	23.6

**Table 8:** Comparative experiments of the proposed method.

Algorithm	Rotation Error RE/(°)	Reprojection Translation Error TE/(px)	Tracking Success Rate/(%)	Time per Frame/(ms)	Parameters/(M)
LK	4.72	8.64	61.3	12.4	0
STC	3.86	6.91	70.5	18.7	0
LightGlue-ORB	2.41	4.27	83.2	24.1	12.6
SuperGlue-SIFT	2.15	3.82	86.7	35.6	24.8
E-LoFTR	2.28	4.03	84.9	31.2	19.3
RGBTrack	2.64	4.65	81.4	27.5	15.1
PixTrack	2.07	3.61	87.5	48.3	33.7
Improved-RBOT	2.83	4.88	79.6	21.3	0.8
Deep-SLAM	2.01	3.42	88.3	29.2	8.7
XRDSLAM	1.92	3.26	89.2	33.5	15.2
Our	1.86	3.14	90.1	19.5	0

**Table 9:** Quantitative comparison results of each algorithm in highly dynamic environments.

Algorithm	Urban Dynamic Scenes RE/(°)	Urban Dynamic Scenes TE/(px)	Urban Dynamic Scenes Success Rate/(%)	Highly Active Indoor Scenes RE/(°)	Highly Active Indoor Scenes TE/(px)	Highly Active Indoor Scenes Success Rate/(%)
LK	7.42	12.63	33.5	7.86	13.15	30.2
STC	5.86	10.47	44.1	6.23	10.84	41.5
LightGlue-ORB	3.87	6.41	68.2	4.15	6.88	64.7
SuperGlue-SIFT	3.64	6.05	70.6	3.91	6.42	67.3
E-LoFTR	3.52	5.83	71.8	3.76	6.21	68.5
RGBTrack	4.08	6.92	65.4	4.33	7.37	62.1
PixTrack	3.75	6.26	69.1	4.02	6.70	66.0
Improved-RBOT	3.93	6.54	66.8	4.21	7.05	63.4
Deep-SLAM	3.31	5.57	74.3	3.57	5.94	71.2
XRDSLAM	3.14	5.22	76.5	3.38	5.61	73.6
Our	2.37	3.84	83.4	2.61	4.23	80.1

**Table 10:** Quantitative comparison results of different algorithms under extreme conditions.

Algorithm	Insufficient Lighting RE/(°)	Insufficient Lighting TE/(px)	Insufficient Lighting Success Rate/(%)	Rapid Motion RE/(°)	Rapid Motion TE/(px)	Rapid Motion Success Rate/(%)	Severe Occlusion RE/(°)	Severe Occlusion TE/(px)	Severe Occlusion Success Rate/(%)
LK	6.85	11.32	42.6	7.92	13.58	35.8	8.56	15.21	28.4
STC	5.21	9.65	51.3	6.14	10.92	46.2	7.03	12.45	39.7
LightGlue-ORB	3.44	5.73	74.1	4.06	6.94	67.3	4.52	7.81	62.5
SuperGlue-SIFT	3.26	5.41	76.4	3.83	6.57	69.6	4.27	7.36	65.2
E-LoFTR	3.11	5.22	77.2	3.71	6.32	70.5	4.11	7.14	66.4
RGBTrack	3.62	6.08	72.3	4.25	7.26	64.8	3.94	6.77	68.1
PixTrack	3.38	5.64	74.8	3.97	6.79	68.2	3.66	6.28	71.3
Improved-RBOT	3.51	5.87	73.5	4.12	7.03	66.1	3.79	6.45	69.7
Deep-SLAM	3.02	5.14	78.5	3.68	6.25	72.4	4.15	7.32	68.9
XRDSLAM	2.86	4.89	80.2	3.42	5.91	74.8	3.89	6.85	71.2
Our	2.15	3.56	86.7	2.48	4.02	82.3	2.96	4.68	78.5

**Table 11:** Algorithm performance on different hardware platforms.

Hardware Platform	Hardware Configuration	Average Time per Frame (ms)	Average Frame Rate (fps)	Average CPU Utilization (%)
PC	Intel i5-10400, CPU only	19.5	51.3	38
Embedded board	Jetson Nano, 4-core A57, GPU disabled	36.8	27.2	72
Android mobile	ARM big.LITTLE architecture, CPU only	48.3	20.7	84

**Table 12:** Results of ablation experiments.

Experiment Group	A	B	C	D	RE/(°)	TE/(px)	Tracking Success Rate/(%)	Time per Frame/(ms)
Baseline	×	×	×	×	4.23	7.86	63.7	27.6
A	√	×	×	×	3.11	5.72	74.2	22.3
A + B	√	√	×	×	2.47	4.45	81.5	20.8
A + B + C	√	√	√	×	2.13	3.78	86.3	20.1
Our	√	√	√	√	1.86	3.14	90.1	19.5

## Data Availability

The data are contained within this article.
